# ExNCDIA: an explainable ensemble learning framework for predicting non-chemotherapy drug-induced agranulocytosis and interpreting structural associations

**DOI:** 10.1039/d6ra06159a

**Published:** 2026-11-05

**Authors:** Xiaojie Huang, Shusong Jiang, Peineng Liu

**Affiliations:** a Department of Pharmacy, Jieyang People's Hospital Jieyang 522000 China huangxj46@alumni.sysu.edu.cn

## Abstract

Non-chemotherapy drug-induced agranulocytosis (NCDIA) is a rare but life-threatening idiosyncratic toxicity whose mechanisms remain largely unresolved; for a subset of drugs, however, the risk has been linked to chemical structure through bioactivation, whereby reactive intermediates injure neutrophils and myeloid progenitors, and haptenate proteins to trigger immune-mediated destruction. Yet the structural features underlying this liability remain poorly defined, and interpretable models for flagging it during design are scarce. Here we report ExNCDIA, an explainable machine learning framework that predicts NCDIA liability directly from chemical structure. From a curated set of 906 drugs (371 NCDIA-positive and 535 NCDIA-negative), we benchmarked complementary open-source representations and imbalance-aware ensemble classifiers, and retained two balanced random forest models—one trained on RDKit molecular descriptors refined by genetic-algorithm feature selection (test ROC-AUC 0.850, MCC 0.591), the other on MACCS substructure keys (test ROC-AUC 0.847, MCC 0.518). Both showed balanced discrimination in training-set cross-validation and on the held-out test set. Crucially, Shapley additive explanations (SHAP) applied to these two non-overlapping representations converged on the same medicinal-chemistry signal: NCDIA risk rises with sulfur-containing pharmacophores, multiple aromatic rings, and nitrogen-bearing/arylamine cores—motifs prone to cytochrome P450- and myeloperoxidase-mediated oxidation to reactive metabolites—and falls with aliphatic, saturated-oxygenated scaffolds lacking such bioactivatable groups. Delivered as a freely accessible web tool, ExNCDIA couples quantitative prediction with interpretable structural associations, supporting early candidate prioritization and post-marketing risk assessment.

## Abstract 2

ExNCDIA predicts non-chemotherapy drug-induced agranulocytosis while identifying bioactivation-associated structural features through explainable machine learning.

## Graphical abstract

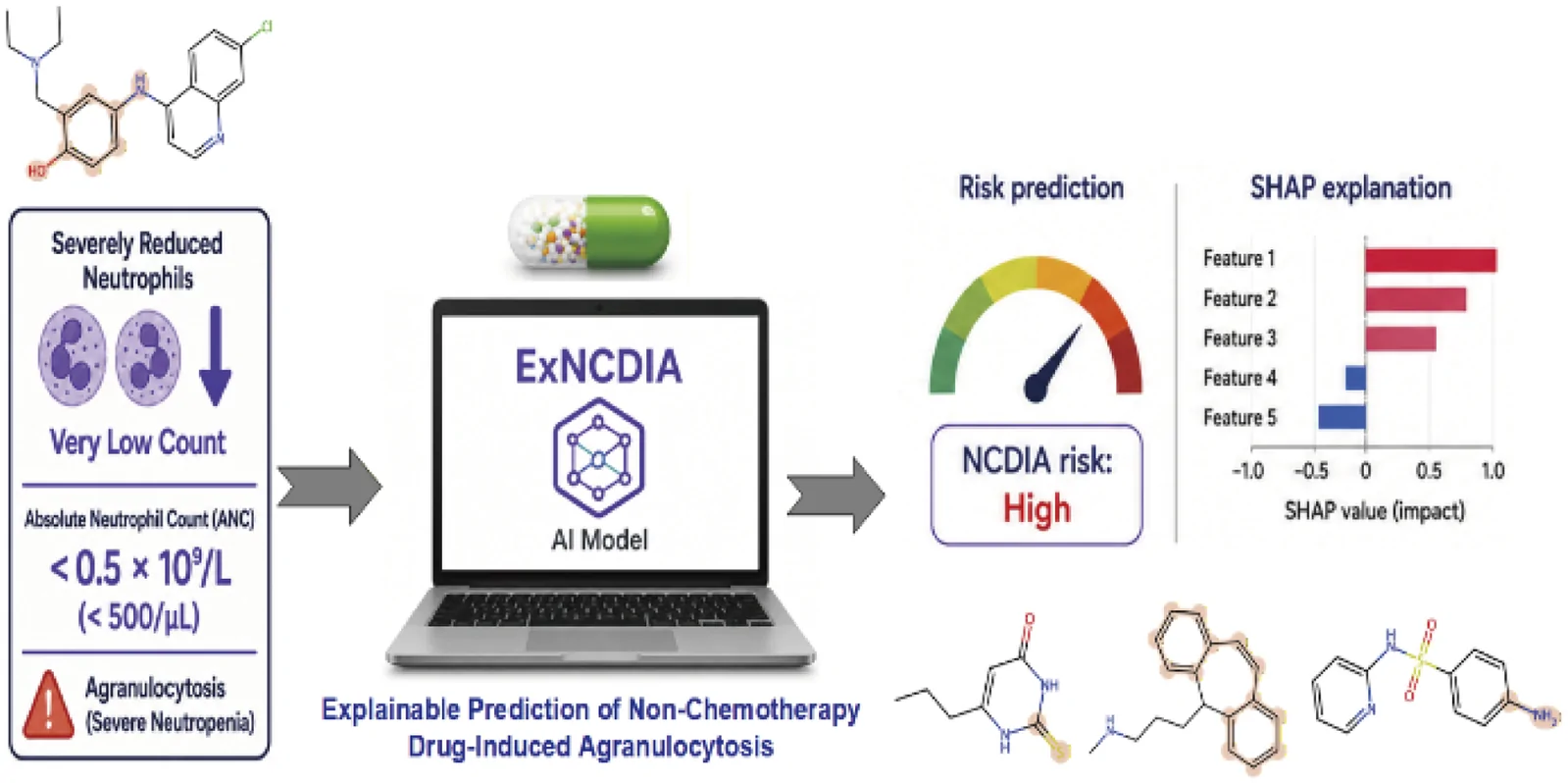

## 1. Introduction

Agranulocytosis is a severe hematological adverse reaction characterized by a marked reduction in circulating neutrophils, typically defined as an absolute neutrophil count below 0.5 × 10^9^ L^−1^.^1,2^ Although uncommon, it can rapidly progress to serious infection, sepsis, and death. Drug exposure is the leading cause of agranulocytosis, accounting for approximately 70–97% of all cases.^3,4^ Non-chemotherapy drug-induced agranulocytosis (NCDIA) represents a particularly challenging subtype because of its low incidence, unpredictable onset, and often unclear dose–response relationship.^4,5^

Unlike chemotherapy-induced neutropenia, which arises predominantly from dose-dependent bone-marrow suppression, NCDIA is thought to proceed through heterogeneous and still incompletely resolved mechanisms, encompassing immune-mediated neutrophil destruction and direct toxicity toward myeloid precursors.^4,6,7^ Among the proposed routes, metabolic bioactivation of the parent drug to reactive intermediates has attracted the most mechanistic attention:^8,9^ for a number of implicated agents, exemplified by clozapine, oxidation by cytochrome P450 enzymes and the neutrophil myeloperoxidase (MPO) system generates electrophilic species that covalently modify cellular proteins, injuring neutrophils and their progenitors and, through hapten formation, priming immune-mediated destruction.^9,10^ Such observations indicate that, at least for a subset of drugs, the liability is partly encoded in molecular structure and its susceptibility to bioactivation. To date, however, this evidence has accrued largely on a compound-by-compound basis, and the structural determinants that generalize across the chemically diverse NCDIA space have not been systematically delineated—a gap that structure-based, interpretable modeling is well positioned to address.

*In silico* toxicity prediction offers a practical route to interrogate such structure-encoded liabilities during drug development and post-marketing evaluation, particularly for rare adverse reactions that are difficult to capture through conventional experimental or epidemiological approaches.^11–13^ Molecular descriptors and fingerprints convert chemical structures into machine-readable features that capture physicochemical properties, topology, and substructural motifs relevant to toxicity.^14,15^ For NCDIA, an initial structure-based machine-learning model demonstrated the feasibility of predicting this endpoint from molecular structure.^16^ Nevertheless, that proof-of-concept work also underscored broader needs that remain important for the field. Because NCDIA-positive evidence is dispersed across systematic reviews, case reports, and regulatory drug-label annotations, the structural basis of this adverse reaction has not yet been comprehensively characterized across a broad clinically annotated drug space. Integrating public adverse-reaction resources such as the Side Effect Resource (SIDER),^17^ which links marketed drugs to side effects extracted from drug labels, may therefore provide complementary coverage of clinically documented NCDIA-associated chemical space and support more systematic structure-toxicity analysis.

Reproducibility, interpretability, and usability are equally important for translating NCDIA prediction into medicinal chemistry and drug-safety practice. Models based on proprietary descriptors or opaque decision rules can be difficult for other researchers to reproduce, validate, or mechanistically interrogate. In contrast, open-source molecular representations, including RDKit descriptors,^18^ Mordred descriptors,^19^ MACCS keys,^20^ and extended-connectivity fingerprints with diameter 4 (ECFP4),^21^ provide transparent and reproducible feature spaces for structure-based modeling. Beyond reproducibility, predictive performance alone is insufficient for decision-making in idiosyncratic toxicity. Medicinal chemists and safety researchers need to know not only whether a compound is predicted to be high risk, but also which molecular properties or structural motifs drive that prediction. Explainable machine learning, particularly Shapley additive explanations (SHAP), can quantify feature-level contributions to model output and help relate data-driven predictions to plausible structure-toxicity hypotheses.^22,23^ Moreover, the absence of an accessible online predictor constrains practical use in both early-stage candidate screening and post-marketing risk assessment, where medicinal chemists and safety researchers require rapid evaluation of molecules without specialized computational expertise.

Building on this rationale, we developed ExNCDIA, an explainable ensemble machine-learning framework for interpreting structural features associated with model predictions. The present study extends previous structure-based modeling of NCDIA in four clinically and chemically relevant directions. First, we assembled an expanded and rigorously curated dataset by incorporating SIDER agranulocytosis-annotated drugs with literature-derived NCDIA evidence, thereby broadening the clinically documented chemical space used for model development. Second, ExNCDIA relies exclusively on open-source molecular representations, including RDKit and Mordred descriptors, MACCS keys, and ECFP4 fingerprints, to enhance transparency and reproducibility. Third, we integrated SHAP-based global and local interpretation with applicability-domain analysis to identify molecular properties and structural motifs driving NCDIA predictions, assess the reliability boundary of predictions, and support mechanistic interpretation of structure-toxicity relationships. Fourth, the optimized models were deployed as a freely accessible online prediction platform (https://exncdia-predictor.streamlit.app/), enabling rapid molecule-level assessment without specialized computational infrastructure. To promote transparency and reuse, the complete dataset, source code, and trained models are openly available at https://github.com/Huangxiaojie2024/ExNCDIA. A schematic representation of the ExNCDIA framework is presented in Fig. 1.

**Fig. 1:**
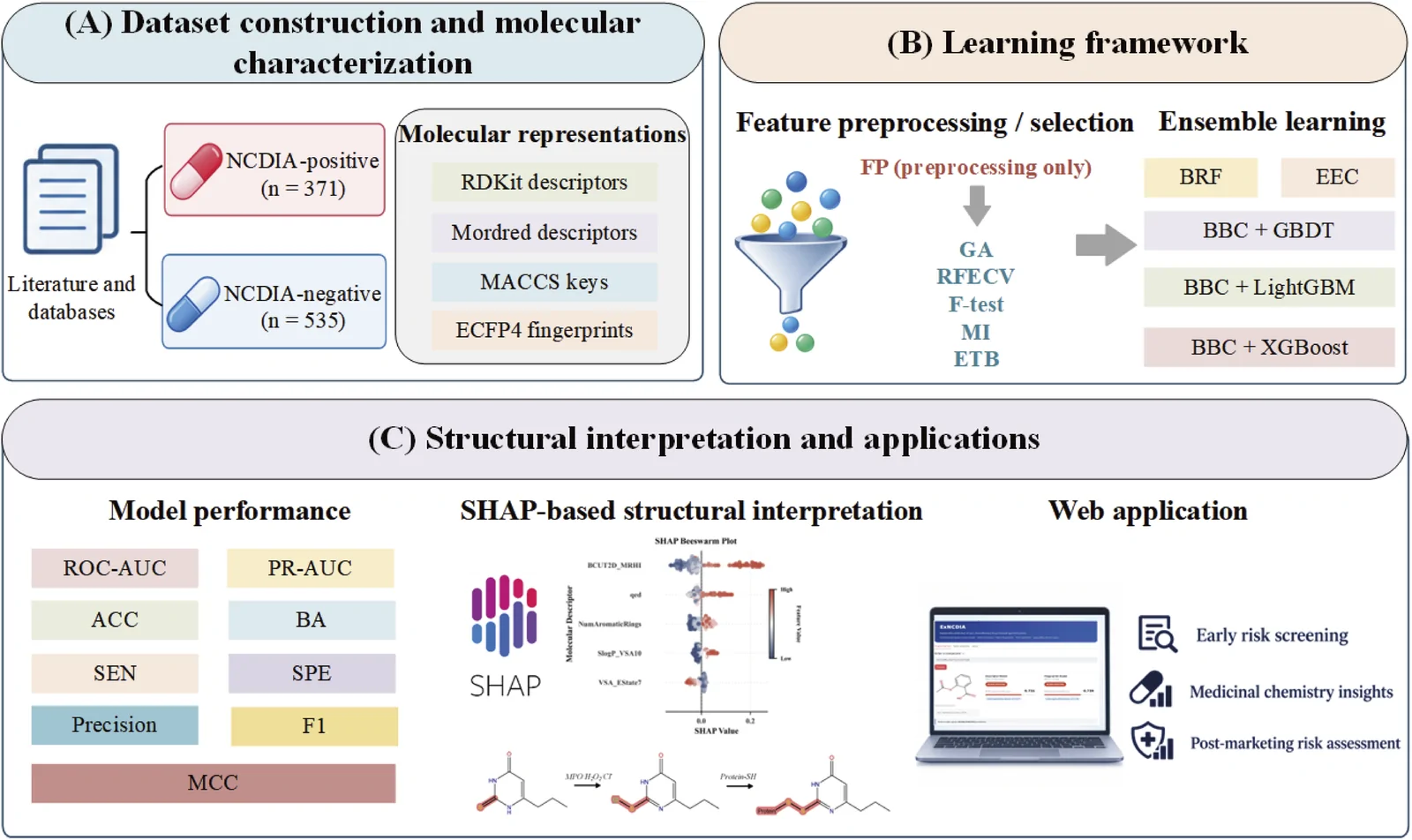
Overview of the ExNCDIA framework for explainable prediction of NCDIA. (A) Dataset construction and molecular characterization. (B) Learning framework, including feature preprocessing and selection, followed by imbalance-aware ensemble learning. (C) Structural interpretation and applications, including SHAP-based structural interpretation and deployment of the web-based prediction application.

## 2. Materials and methods

### 2.1. Methodological overview

As illustrated in Fig. 1, the ExNCDIA workflow comprises three core components: (1) dataset construction and molecular characterization, in which a curated chemical library with operational NCDIA class labels is assembled and represented using four complementary molecular feature sets; (2) learning framework, integrating systematic feature preprocessing, multi-strategy feature selection, imbalance-aware ensemble modelling, and performance validation; and (3) structural interpretation and application, employing SHAP to characterize molecular features contributing to NCDIA predictions at both global and local levels and to relate the identified structural associations to mechanistic context, culminating in deployment of the final models as a publicly accessible web-based predictor. This integrated design connects predictive performance with chemically meaningful structural interpretation, thereby supporting early-stage candidate screening and post-marketing risk assessment.

### 2.2. Data collection and preparation

Drug–adverse-event associations were obtained from SIDER (version 4.1),^17^ which maps adverse events extracted from regulatory product labels to MedDRA terms. For model development, two operational class labels were used. Drugs with documented evidence of NCDIA were designated NCDIA-positive, whereas drugs not annotated as NCDIA-associated in the sources examined after application of the exclusion and cross-checking procedures described below were designated NCDIA-negative. The NCDIA-positive class was constructed by combining (1) drugs from our previously curated dataset,^16^ which was originally compiled from published systematic reviews and case reports, and (2) drugs with agranulocytosis annotations in SIDER.

To construct the candidate NCDIA-negative class and reduce potential false-negative labels, we applied a deliberately broad, prespecified hematologic exclusion dictionary. Any SIDER drug annotated with at least one term related to agranulocytosis, neutropenia (including febrile neutropenia), leukopenia, or a quantitative or qualitative abnormality in white blood cell or neutrophil counts was removed from the candidate NCDIA-negative pool. These terms were used solely to exclude potentially ambiguous drugs from the candidate NCDIA-negative pool and were not considered sufficient for an NCDIA-positive assignment. Candidate NCDIA-negative drugs were additionally cross-checked against the literature sources used to construct the NCDIA-positive class. When qualifying evidence of NCDIA was identified in either the literature or SIDER, the NCDIA-positive assignment took precedence. Drugs with unresolved label conflicts or ambiguous class assignments were excluded from model development. Chemotherapy agents classified under Anatomical Therapeutic Chemical code L01 were subsequently removed to avoid confounding by expected dose-dependent myelosuppression.

To ensure data quality, the collected compounds were subjected to a four-step curation protocol using the RDKit cheminformatics toolkit (version 2022.09.5):^18^ (1) SMILES strings were parsed and desalted by retaining the largest covalent fragment, after which compounds containing metal atoms were excluded to remove inorganic and organometallic entities; (2) the remaining structures underwent valence and bond-order normalization, neutralization of formal charges, canonical tautomer selection, and removal of stereochemical information consistent with the two-dimensional molecular representations used in this study; (3) canonical SMILES were generated, residual multi-component entries were discarded, and compounds with molecular weight outside the 30–1000 Da range were filtered out; (4) duplicate entries were resolved by InChIKey matching with canonical SMILES as a fallback, and records assigning conflicting class labels to the same structure were manually adjudicated to retain a single final assignment.

The final curated collection comprised 906 drugs assigned operational NCDIA class labels (371 NCDIA-positive and 535 NCDIA-negative), which were partitioned into a training set (*n* = 724) and a held-out test set (*n* = 182) at an 8 : 2 ratio *via* stratified random sampling, thereby preserving the class proportions across both subsets (Table 1). Compound-level entries with standardized SMILES and NCDIA labels are provided in Tables S1 and S2, and the complete dataset has been deposited at https://github.com/Huangxiaojie2024/ExNCDIA.

**Table 1:** Summary of the datasets utilized in this study

Datasets	NCDIA-positive	NCDIA-negative	Total
Training set	296	428	724
Test set	75	107	182
Total	371	535	906

### 2.3. Molecular representation

Numerical encoding of chemical structure constitutes the foundation of structure-based toxicity prediction. To capture diverse aspects of molecular identity, four complementary feature sets were computed using open-source Python cheminformatics packages and evaluated independently. RDKit molecular descriptors (version 2022.09.5, 208 features) were calculated to quantify physicochemical, topological, electronic, and fragment-related properties of drug molecules, including functional group and substructural motif counts relevant to bioactivation-mediated toxicity.^18^ Mordred descriptors (version 1.2.0, 1613 features) provide a large-scale open-source descriptor set spanning constitutional, topological, autocorrelation, and electronic domains.^19^ MACCS keys (166 binary bits), originally developed for rapid substructure searching, encode the presence or absence of predefined structural patterns commonly used in drug discovery.^20^ Although these keys allow straightforward identification of predefined substructures, they may not fully capture the positional and chemical context of matched functional groups, and potentially relevant structural motifs outside the predefined key set remain unrepresented. In addition, 2048-bit ECFP4 representations (Morgan radius = 2) were generated to encode atom-centred circular environments extending up to two bonds from each atom.^21^ Feature preprocessing and selection were performed independently for each feature set, enabling systematic comparison of their individual predictive value for NCDIA risk. Collectively, these representations provided broad coverage of molecular physicochemical properties and structural features relevant to structure-based toxicity modeling.

### 2.4. Learning framework

#### 2.4.1. Feature preprocessing and selection

Raw molecular features often contain missing values, irrelevant variables, redundant information, and differences in scale, all of which can compromise model robustness if not properly addressed.^24^ A four-step feature preprocessing (FP) pipeline was therefore applied to each feature set: (1) removal of features containing missing values to guarantee complete feature vectors; (2) *z*-score standardization of the molecular descriptors (RDKit and Mordred) to place continuous descriptors on a common numerical scale—this step was omitted for the binary MACCS and ECFP4 fingerprints, whose bits already lie on a common 0/1 scale; (3) variance thresholding to discard zero-variance features that carry no discriminative information; and (4) Pearson correlation pruning, where applicable, in which one feature was removed from each pair exhibiting an absolute correlation greater than 0.9, thereby mitigating multicollinearity.

Following preprocessing, five complementary feature selection strategies were applied to identify informative feature subsets from different algorithmic perspectives: F-test, mutual information (MI), recursive feature elimination with cross-validation (RFECV), genetic algorithm (GA), and embedded tree-based feature selection (ETB). Together, these strategies span filter-based (F-test and MI), wrapper-based (RFECV and GA), and embedded (ETB) paradigms, enabling a systematic comparison to identify the most effective selection strategy for each molecular representation.

F-test^25^ applies analysis of variance to evaluate the univariate statistical association between each individual feature and the binary class label. Features with *p*-values below 0.05 were retained to enrich variables with significant single-feature discrimination between NCDIA-positive and NCDIA-negative compounds.

MI^26^ quantifies both linear and nonlinear statistical dependencies between each feature and the class label; features with MI scores greater than zero were retained.

RFECV^27^ iteratively eliminates the least important features and automatically determines the optimal feature subset by monitoring cross-validation performance.

GA^28^ performs evolutionary optimization over candidate feature subsets, evolving a population of binary feature masks *via* tournament selection (tournament size = 3), two-point crossover, and bit-flip mutation. Hyperparameters were set as follows: population size = 50, generations = 40, crossover probability = 0.5, per-individual mutation probability = 0.2, and per-gene bit-flip probability = 0.05.

ETB^29^ uses model-derived feature importance from a Balanced Random Forest (BRF)^30^ model to identify informative variables; features with importance values ≥0.001 were preserved.

For the wrapper- and embedded-based methods (RFECV, GA, and ETB), a BRF classifier served as the base estimator, whereas the filter methods (F-test and MI) are estimator-independent. The feature subset retained by each of the five strategies was then evaluated with a BRF classifier by 10-fold cross-validation on the training set, using the Matthews correlation coefficient (MCC) as the primary metric for its robustness on imbalanced datasets.^31^ For every performance metric, the ten fold-specific estimates were summarized as the mean ± sample standard deviation (SD).

#### 2.4.2. Imbalance-aware ensemble modeling and validation

The training set was moderately imbalanced: NCDIA-positive drugs (*n* = 296, 40.9%) were outnumbered by NCDIA-negative drugs (*n* = 428, 59.1%), yielding a class ratio of 1 : 1.45. Even at this moderate skew, classifiers trained directly on such data can still bias their decision boundary toward the majority class and thereby under-detect the clinically critical minority cases.^32,33^ Accordingly, we evaluated both imbalance-aware ensembles and simpler algorithm-matched baselines. The imbalance-aware estimators were implemented with imbalanced-learn (version 0.12.3).^34^

The five imbalance-aware classifiers comprised balanced random forest (BRF), easy ensemble classifier (EEC), and balanced bagging classifier (BBC) combined separately with gradient boosting decision tree (GBDT),^35^ light gradient boosting machine (LightGBM),^36^ or extreme gradient boosting (XGBoost).^37^ BRF balances the bootstrap sample used for each tree;^30^ EEC combines AdaBoost learners fitted to repeatedly undersampled majority-class subsets;^38^ and BBC rebalances each bootstrap replicate before fitting its base learner.^39^ To provide simpler comparators and isolate the effect of imbalance handling, we additionally evaluated standard and class-weighted versions of random forest (RF), AdaBoost, GBDT, LightGBM, and XGBoost, giving 15 classifiers per feature subset. Balanced class weights, or the corresponding observation weights supported by each implementation, were calculated from the training portion of each fold. RF, GBDT and AdaBoost were implemented in scikit-learn (version 1.5.1), LightGBM in lightgbm (version 3.3.5), and XGBoost in xgboost (version 1.6.1).

Hyperparameters were tuned with Hyperopt (version 0.2.7),^40^ using the tree-structured Parzen estimator to optimize mean Matthews correlation coefficient (MCC) in 10-fold cross-validation of the training set. MCC was selected as the optimization objective because it incorporates all four outcomes of the confusion matrix and remains informative under class imbalance.^31^ Model selection was primarily based on cross-validated MCC, with sensitivity and the absolute sensitivity – specificity difference used as secondary criteria when MCC values were similar. These selection criteria were applied exclusively to the training-set cross-validation results before examination of held-out test-set performance. The selected classifiers were subsequently refitted on the complete training set and evaluated on the held-out test set.

As an additional structure-similarity baseline, an ECFP4-Tanimoto similarity-weighted *k*-nearest-neighbour (kNN) classifier was evaluated using the same fixed training-test split. The original 2048-bit ECFP4 fingerprints were used to calculate pairwise Tanimoto similarities. For each query compound, the probability of being NCDIA-positive was calculated as the Tanimoto-similarity-weighted proportion of NCDIA-positive compounds among its *k* nearest neighbours in the training set. Candidate values of *k* (1, 3, 5, 7, 9, 11, 15, and 21) were compared exclusively within the training set using shuffled stratified 10-fold cross-validation, with mean MCC as the selection criterion. The value of *k* selected by training-set cross-validation was then fixed before evaluation on the held-out test set, with a probability threshold of 0.50 used for class assignment.

Predictive performance was first characterized by 10-fold cross-validation on the training data and subsequently examined on the held-out test set. The cross-validation results were used to select the final representation-classifier combinations, whereas the held-out test set was used to evaluate post-selection predictive performance and to compare model predictions on the same compounds. Performance was evaluated using nine indicators: area under the receiver operating characteristic curve (ROC-AUC), area under the precision-recall curve (PR-AUC), accuracy (ACC), balanced accuracy (BA), sensitivity (SEN), specificity (SPE), precision, F1 score, and Matthews correlation coefficient (MCC). ROC-AUC and PR-AUC were threshold-independent metrics; PR-AUC was calculated by trapezoidal integration of the precision-recall curve and was included because it is particularly informative when the positive class is less prevalent. Threshold-dependent metrics were calculated from model-predicted class labels. True positives (TP) and true negatives (TN) denote correctly classified NCDIA-positive and NCDIA-negative drugs, respectively; false positives (FP) are NCDIA-negative drugs classified as NCDIA-positive, whereas false negatives (FN) are NCDIA-positive drugs classified as NCDIA-negative. Here, FP denotes false positives and is distinct from the feature-preprocessing abbreviation used in Section 2.4.1.1
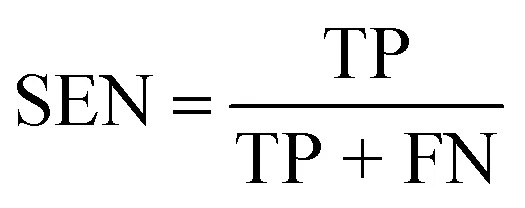
2
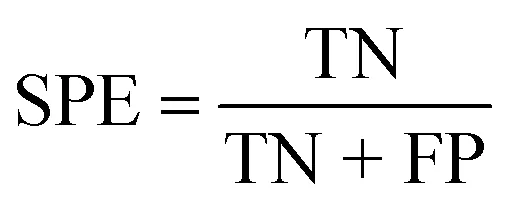
3
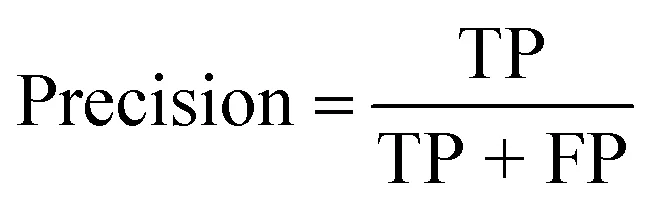
4
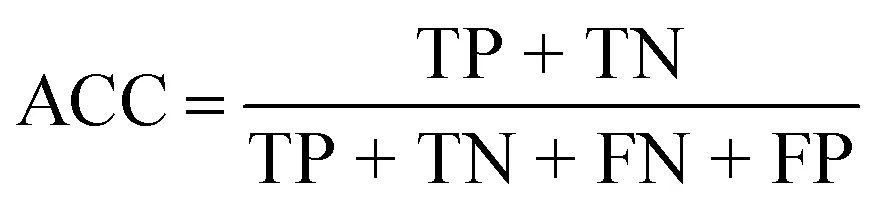
5
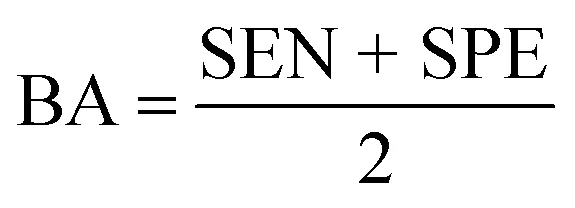
6
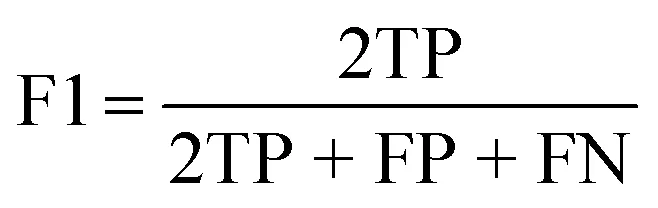
7
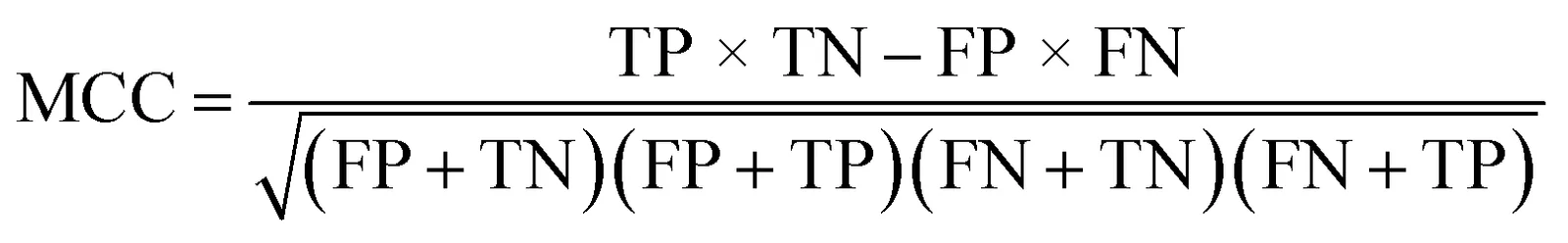


SEN and SPE quantify performance for the NCDIA-positive and NCDIA-negative classes, respectively; BA is their arithmetic mean. Precision measures the proportion of predicted-positive compounds that are NCDIA-positive, and F1 is the harmonic mean of precision and SEN. MCC summarizes the complete confusion matrix as a correlation-like score bounded between −1 and +1 and remains informative when class sizes differ.^31,41^ Cross-validation results are reported as mean ± SD across the ten folds. For held-out test-set metrics, percentile-based 95% confidence intervals (CIs) were obtained from 5000 stratified bootstrap resamples, with the NCDIA-positive and NCDIA-negative classes sampled separately with replacement to preserve class counts. Differences between models on the same test compounds were assessed by paired stratified bootstrap resampling using identical resampled compound indices for each model; a 95% CI for a paired difference that included zero was not interpreted as evidence of a clear difference. Owing to space constraints, the main text reports ROC-AUC, BA, SEN, SPE, and MCC for the five imbalance-aware models; complete results for all nine metrics, all baselines, and the paired comparisons are provided in the SI.

### 2.5. Applicability domain assessment

Predictions from a machine-learning model are most appropriately interpreted for query compounds that fall within the chemical space represented by its training data; quantifying this boundary—the applicability domain (AD)—is a core requirement of the OECD principles for (quantitative) structure–activity relationship (Q)SAR model validation.^42^ We delineated the AD with the Euclidean Applicability Domain 1.0 software,^43^ which quantifies structural (dis)similarity *via* Euclidean distances within the modeling feature space. For every molecule the program returns a normalized mean distance score calibrated to the training set, where 0 marks the most representative compound and 1 the most peripheral. The 0–1 interval spanned by the training compounds thus constitutes the model's AD: a drug whose score falls within this interval is regarded as structurally supported by the training data, whereas a score exceeding 1 identifies a compound lying beyond the learned chemical space and therefore represents an extrapolative prediction.

### 2.6. Multi-level model interpretation *via* SHAP

To complement predictive performance with model interpretation, we performed a post hoc SHAP analysis to characterize the structural and physicochemical features contributing to NCDIA predictions. SHAP is a game-theoretic framework that assigns each feature a quantitative additive contribution to the model output, enabling both global and individual-level interpretation.^22^

Before interpretation, the robustness of the SHAP results was assessed using the training data across alternative preprocessing/feature-selection settings and 10 cross-validation folds. For each final model, we examined whether its five leading SHAP features remained within the corresponding Top-20 lists. Consistency was further quantified using Spearman correlations between SHAP rankings and Jaccard indices for Top-20 feature overlap, with higher values indicating greater SHAP stability.

Using the final classifiers fitted on the complete training set, SHAP-based interpretation was subsequently performed on the held-out test set, proceeding from the overall test population to individual compounds. At the global level, mean absolute SHAP values were used to rank features by their overall importance, whereas signed SHAP values and feature-level attribution patterns were examined to determine whether individual features were associated with higher or lower NCDIA-positive model outputs. The interpretation approach was adapted to the type of molecular representation used by each selected model. For selected models based on binary fingerprint features, SHAP contributions were compared between the present and absent states of individual bits to identify structural patterns associated with higher or lower NCDIA-positive predictions. For selected models based on continuous molecular descriptors, SHAP dependence plots were used to characterize the relationship between descriptor values and their contributions to the model output, including nonlinear trends and approximate value ranges over which the direction of contribution changed. At the local level, SHAP waterfall plots decomposed individual predictions into feature-level contributions, clarifying which features contributed toward assignment to the NCDIA-positive or NCDIA-negative class.

All SHAP computations were carried out in Python using the SHAP library (version 0.46.0).

### 2.7. Web-based platform implementation

The final models identified through the benchmarking described above were selected for public deployment. To make these predictors usable beyond a local computational environment, we packaged them as an open-access web application using Streamlit, a Python framework that wraps analytical scripts into interactive browser interfaces without separate front-end development. The serialized models were embedded into a server-side routine that accepts a SMILES string as input, computes the relevant molecular features on the fly, and—for each deployed model—returns the predicted NCDIA class and an NCDIA-positive model score, evaluates the query against its previously established applicability domain, and generates a SHAP waterfall plot of the prediction's feature contributions. The application is hosted on Streamlit Community Cloud and accessible through any standard web browser.

## 3. Results and discussion

### 3.1. Chemical space and scaffold diversity of the NCDIA dataset

The curated dataset comprised 906 drugs assigned operational NCDIA class labels, of which 371 (40.9%) were NCDIA-positive and 535 (59.1%) were NCDIA-negative (Table 1). Following a stratified random split, 724 drugs (296 NCDIA-positive; 428 NCDIA-negative) were assigned to the training set and 182 drugs (75 NCDIA-positive; 107 NCDIA-negative) to the test set, preserving the class composition at 1 : 1.45 in the training set and 1 : 1.43 in the test set. Compound-level entries for the two subsets are provided in Tables S1 and S2, respectively, and the complete dataset has been deposited at https://github.com/Huangxiaojie2024/ExNCDIA.

The chemical-space coverage of the dataset was first examined in the molecular weight (*M*_W_) *versus* calculated octanol-water partition coefficient (ALogP) plane (Fig. 2A). The training and test sets occupied an overlapping region centered within the drug-like chemical space, with the majority of compounds falling between *M*_W_ 150 and 500 Da and ALogP between −2 and 5. Test-set compounds were distributed throughout this region rather than clustering near its boundary, consistent with broad overlap between the training and test chemical spaces. Pairwise structural similarity across the complete dataset, quantified using ECFP4 Tanimoto coefficients (Fig. 2B), was low (mean ± SD = 0.097 ± 0.052; median = 0.091), consistent with broad structural heterogeneity and limited redundancy. At the compound level, the test set was not dominated by close training-set analogues, with a median maximum ECFP4 Tanimoto similarity of 0.449 and 53.8% of test compounds below 0.50 (Fig. S1). The complementary uniform manifold approximation and projection (UMAP) visualization based on ECFP4 Tanimoto distances showed substantial overlap between NCDIA-positive and NCDIA-negative compounds across the main projected region, although some peripheral regions were predominantly occupied by one class; training and test compounds were also distributed across overlapping regions of the projection (Fig. S2).

**Fig. 2:**
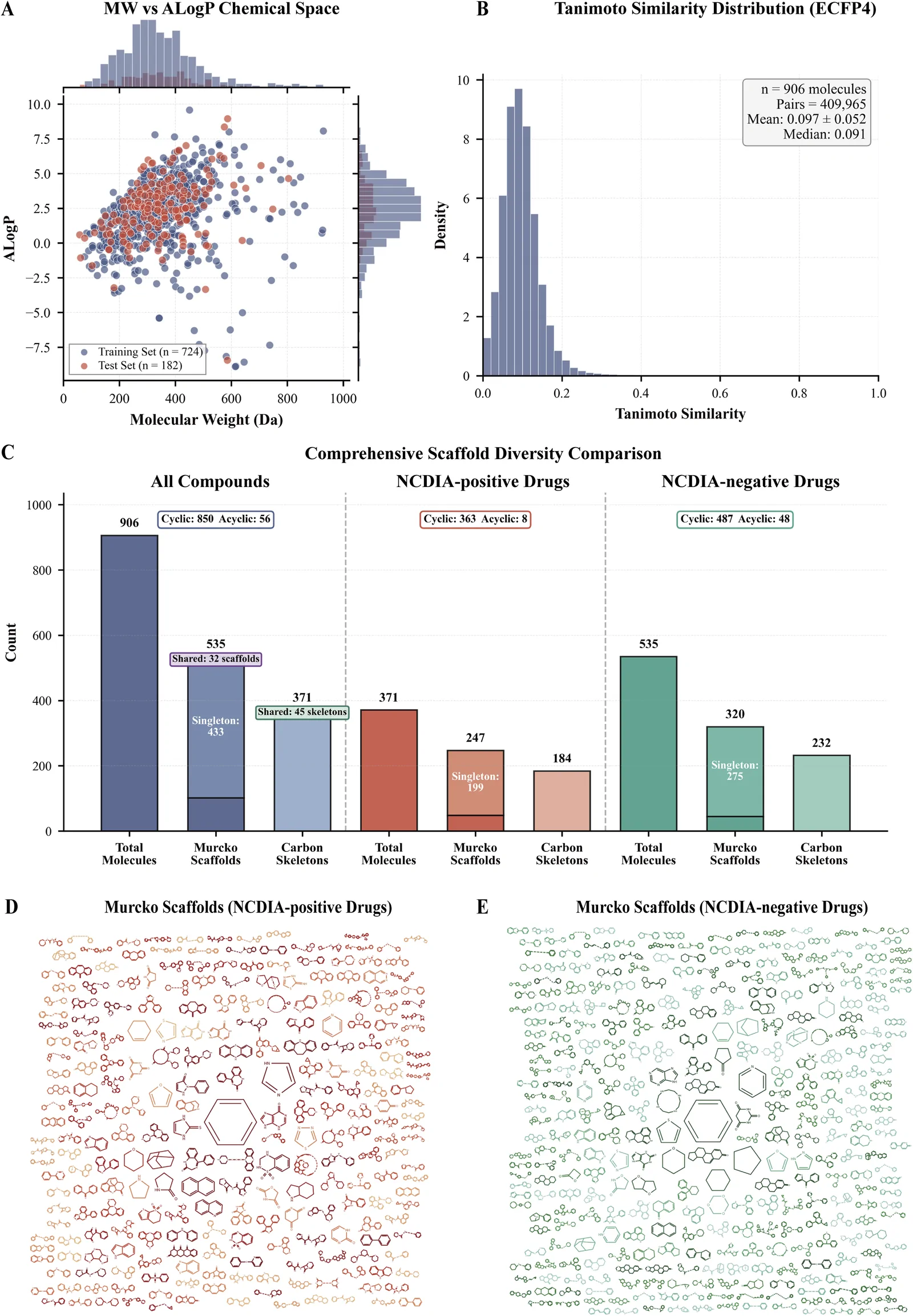
Chemical space and scaffold diversity of the NCDIA dataset. (A) *M*_W_*versus* ALogP chemical space of the training and held-out test sets, with marginal histograms showing the univariate distributions of *M*_W_ and ALogP. (B) Distribution of pairwise ECFP4 Tanimoto similarities across the full dataset. (C) Scaffold-diversity statistics for the full dataset and for the NCDIA-positive and NCDIA-negative classes, reporting total molecule counts (with cyclic/acyclic breakdown), unique Bemis–Murcko scaffolds (with singleton subsets and the number shared between classes), and unique carbon skeletons (with the number shared between classes). (D and E) Galleries of the unique Bemis–Murcko scaffolds of the NCDIA-positive and NCDIA-negative drugs; within each gallery, more frequent scaffolds are rendered larger and positioned closer to the center.

Structural diversity at the scaffold level was further assessed using Bemis–Murcko scaffolds and carbon skeletons (Fig. 2C).^44^ The 906 drugs reduced to 535 unique Murcko scaffolds—433 of which appeared only once (80.9%)—and 371 carbon skeletons, corresponding to a Murcko scaffold diversity of 0.629 (unique scaffolds per ring-containing molecule); cyclic chemotypes accounted for 93.8% of the collection. When the dataset was partitioned by NCDIA outcome, the NCDIA-positive and NCDIA-negative subsets spanned 247 and 320 Murcko scaffolds, respectively, but only 32 Murcko scaffolds and 45 carbon skeletons were common to both classes. This limited overlap—driven in part by the high proportion of singleton scaffolds—indicates limited exact scaffold sharing between the two NCDIA classes. The Bemis–Murcko scaffolds of each class are shown in Fig. 2D and E, with scaffold size and centrality jointly encoding occurrence frequency within the corresponding class.

To complement the scaffold-level characterization, we further examined the functional-group composition of the complete dataset using RDKit fragment definitions (Fig. S3). The two classes differed in the prevalence of several functional groups: NCDIA-positive compounds showed higher frequencies of several nitrogen-containing functionalities, including tertiary and secondary amines, aromatic nitrogen and aniline, as well as sulfur-containing groups such as thioethers, whereas aliphatic hydroxyl, ether, ester and ketone functionalities were more prevalent among NCDIA-negative compounds. NCDIA-positive drugs also contained a greater number of functional-group types per molecule than NCDIA-negative drugs (median 5 *vs.* 4; Mann–Whitney *p* = 2.44 × 10^−12^). These results extend the scaffold analysis by demonstrating broad functional-group coverage together with distinct compositional tendencies between the two NCDIA classes.

Taken together, the chemical-space, scaffold, and functional-group analyses demonstrate that the assembled dataset spans a structurally and compositionally diverse drug space. The two NCDIA classes encompass broad but partially distinct chemotype and functional-group distributions, providing an appropriate chemical-space context for interpreting the molecular features subsequently identified by the predictive models.

### 3.2. Identification of optimal feature subsets for NCDIA prediction

To identify informative representations for NCDIA prediction, four feature sets—RDKit descriptors, Mordred descriptors, MACCS keys, and ECFP4 fingerprints—were each evaluated after FP alone and after five additional selection strategies (F-test, MI, RFECV, GA, and ETB). All evaluations employed a BRF classifier assessed by 10-fold cross-validation on the training set, with MCC as the primary metric. The complete results for all 24 feature set – method combinations, including nine performance metrics reported as mean ± SD, are provided in Table S3; Table 2 summarizes the optimal subset for each representation using the five main-text metrics.

**Table 2:** Optimal feature subset for each molecular representation selected by mean MCC. Performance values are mean ± SD across the 10 cross-validation folds

Feature set	Original no. of features	Feature processing/selection method	No. of retained features	ROC-AUC	BA	SEN	SPE	MCC
RDKit	208	GA	85	0.834 ± 0.049	0.758 ± 0.047	0.749 ± 0.101	0.768 ± 0.078	0.515 ± 0.091
Mordred	1613	GA	216	0.807 ± 0.060	0.738 ± 0.071	0.763 ± 0.084	0.714 ± 0.102	0.472 ± 0.140
MACCS	166	FP	131	0.821 ± 0.042	0.753 ± 0.055	0.752 ± 0.075	0.755 ± 0.085	0.503 ± 0.109
ECFP4	2048	GA	986	0.816 ± 0.049	0.743 ± 0.038	0.675 ± 0.077	0.812 ± 0.100	0.499 ± 0.089

For RDKit descriptors, GA produced the highest mean MCC (0.515 ± 0.091) with 85 retained features and yielded ROC-AUC = 0.834 ± 0.049, BA = 0.758 ± 0.047, SEN = 0.749 ± 0.101, and SPE = 0.768 ± 0.078. ETB ranked second by MCC (0.498 ± 0.117; 110 features), whereas the remaining strategies gave similar mean MCC values (0.480–0.485).

For Mordred descriptors, all strategies produced lower mean MCC values than the corresponding RDKit subsets. GA was optimal by the prespecified criterion (216 retained features), with ROC-AUC = 0.807 ± 0.060, BA = 0.738 ± 0.071, SEN = 0.763 ± 0.084, SPE = 0.714 ± 0.102, and MCC = 0.472 ± 0.140. The mean MCC across the remaining strategies ranged from 0.447 to 0.467.

For MACCS keys, FP without further selection was optimal by mean MCC, retaining 131 keys and yielding ROC-AUC = 0.821 ± 0.042, BA = 0.753 ± 0.055, SEN = 0.752 ± 0.075, SPE = 0.755 ± 0.085, and MCC = 0.503 ± 0.109. Further selection did not improve MCC (0.485–0.493), indicating that the compact predefined key set remained informative after preprocessing.

For the 2048-bit ECFP4 representation, preprocessing retained 1943 bits and GA gave the highest mean MCC, retaining 986 bits and yielding ROC-AUC = 0.816 ± 0.049, BA = 0.743 ± 0.038, SEN = 0.675 ± 0.077, SPE = 0.812 ± 0.100, and MCC = 0.499 ± 0.089. Compared with the preprocessing-only MACCS feature set, the GA-selected ECFP4 feature set exhibited lower SEN (0.675 ± 0.077 *vs.* 0.752 ± 0.075) despite a similar MCC (0.499 ± 0.089 *vs.* 0.503 ± 0.109), indicating less balanced class-wise performance than MACCS_FP. Despite its original design for substructure searching, MACCS achieved predictive performance comparable to ECFP4 in this study. Its predefined structural patterns also facilitate relatively straightforward interpretation, whereas individual bits in the 2048-bit ECFP4 representation may correspond to multiple atomic environments, complicating their interpretation.

Based on cross-validated performance and class-wise balance, the GA-selected RDKit descriptor subset (RDKit_GA) and the preprocessing-only MACCS key subset (MACCS_FP) were selected for subsequent ensemble-model construction; the features comprising each (85 RDKit descriptors and 131 MACCS keys, respectively) are listed in Table S4.

### 3.3. Predictive performance of ensemble models on the selected feature subsets

Following identification of the RDKit_GA and MACCS_FP feature subsets, 15 classifiers were evaluated for each subset: five imbalance-aware ensembles (BRF, EEC, BBC + GBDT, BBC + LightGBM, and BBC + XGBoost) and standard or class-weighted RF, AdaBoost, GBDT, LightGBM, and XGBoost baselines. Training-set cross-validation results for the five imbalance-aware models are summarized in Table 3, and their held-out test-set estimates are presented separately in Table 4. The corresponding test-set ROC curves are shown in Fig. 3. Complete results for all models and their optimized hyperparameters are provided in Tables S5 and S6, respectively.

**Table 3:** Training-set 10-fold cross-validation performance of the five imbalance-aware classifiers. Values are mean ± SD across the ten folds

Feature subset	Model	ROC-AUC	BA	SEN	SPE	MCC
RDKit_GA	BRF	0.834 ± 0.049	0.758 ± 0.047	0.749 ± 0.101	0.768 ± 0.078	0.515 ± 0.091
	EEC	0.830 ± 0.046	0.753 ± 0.057	0.752 ± 0.106	0.754 ± 0.076	0.503 ± 0.107
	BBC + GBDT	0.825 ± 0.057	0.757 ± 0.048	0.711 ± 0.103	0.803 ± 0.079	0.518 ± 0.090
	BBC + LightGBM	0.828 ± 0.044	0.756 ± 0.039	0.725 ± 0.069	0.786 ± 0.081	0.512 ± 0.080
	BBC + XGBoost	0.820 ± 0.049	0.749 ± 0.044	0.741 ± 0.086	0.756 ± 0.078	0.495 ± 0.087
MACCS_FP	BRF	0.821 ± 0.042	0.753 ± 0.055	0.752 ± 0.075	0.755 ± 0.085	0.503 ± 0.109
	EEC	0.815 ± 0.055	0.747 ± 0.037	0.770 ± 0.079	0.723 ± 0.074	0.487 ± 0.072
	BBC + GBDT	0.812 ± 0.049	0.737 ± 0.050	0.694 ± 0.071	0.780 ± 0.086	0.476 ± 0.102
	BBC + LightGBM	0.802 ± 0.045	0.726 ± 0.031	0.702 ± 0.065	0.749 ± 0.079	0.450 ± 0.067
	BBC + XGBoost	0.793 ± 0.041	0.730 ± 0.038	0.732 ± 0.084	0.729 ± 0.084	0.458 ± 0.074

**Table 4:** Held-out test-set performance of the five imbalance-aware classifiers. Values are point estimates with 95% stratified-bootstrap confidence intervals

Feature subset	Model	ROC-AUC	BA	SEN	SPE	MCC
RDKit_GA	BRF	0.850 (0.791–0.903)	0.799 (0.739–0.857)	0.813 (0.720–0.893)	0.785 (0.710–0.860)	0.591 (0.472–0.706)
	EEC	0.845 (0.785–0.898)	0.745 (0.682–0.807)	0.733 (0.640–0.827)	0.757 (0.673–0.832)	0.486 (0.361–0.608)
	BBC + GBDT	0.842 (0.782–0.896)	0.759 (0.696–0.820)	0.760 (0.667–0.853)	0.757 (0.673–0.832)	0.511 (0.388–0.633)
	BBC + LightGBM	0.833 (0.771–0.887)	0.731 (0.664–0.795)	0.733 (0.627–0.827)	0.729 (0.645–0.813)	0.456 (0.324–0.581)
	BBC + XGBoost	0.823 (0.761–0.880)	0.749 (0.683–0.810)	0.760 (0.667–0.853)	0.738 (0.654–0.822)	0.492 (0.361–0.613)
MACCS_FP	BRF	0.847 (0.788–0.899)	0.761 (0.699–0.824)	0.747 (0.653–0.840)	0.776 (0.692–0.850)	0.518 (0.396–0.643)
	EEC	0.823 (0.762–0.880)	0.725 (0.658–0.790)	0.693 (0.587–0.800)	0.757 (0.673–0.832)	0.448 (0.316–0.577)
	BBC + GBDT	0.832 (0.771–0.884)	0.735 (0.668–0.797)	0.693 (0.587–0.800)	0.776 (0.692–0.850)	0.468 (0.337–0.594)
	BBC + LightGBM	0.823 (0.759–0.878)	0.737 (0.669–0.801)	0.707 (0.600–0.800)	0.766 (0.682–0.841)	0.470 (0.338–0.598)
	BBC + XGBoost	0.812 (0.749–0.869)	0.707 (0.639–0.771)	0.667 (0.560–0.773)	0.748 (0.664–0.832)	0.413 (0.277–0.541)

**Fig. 3:**
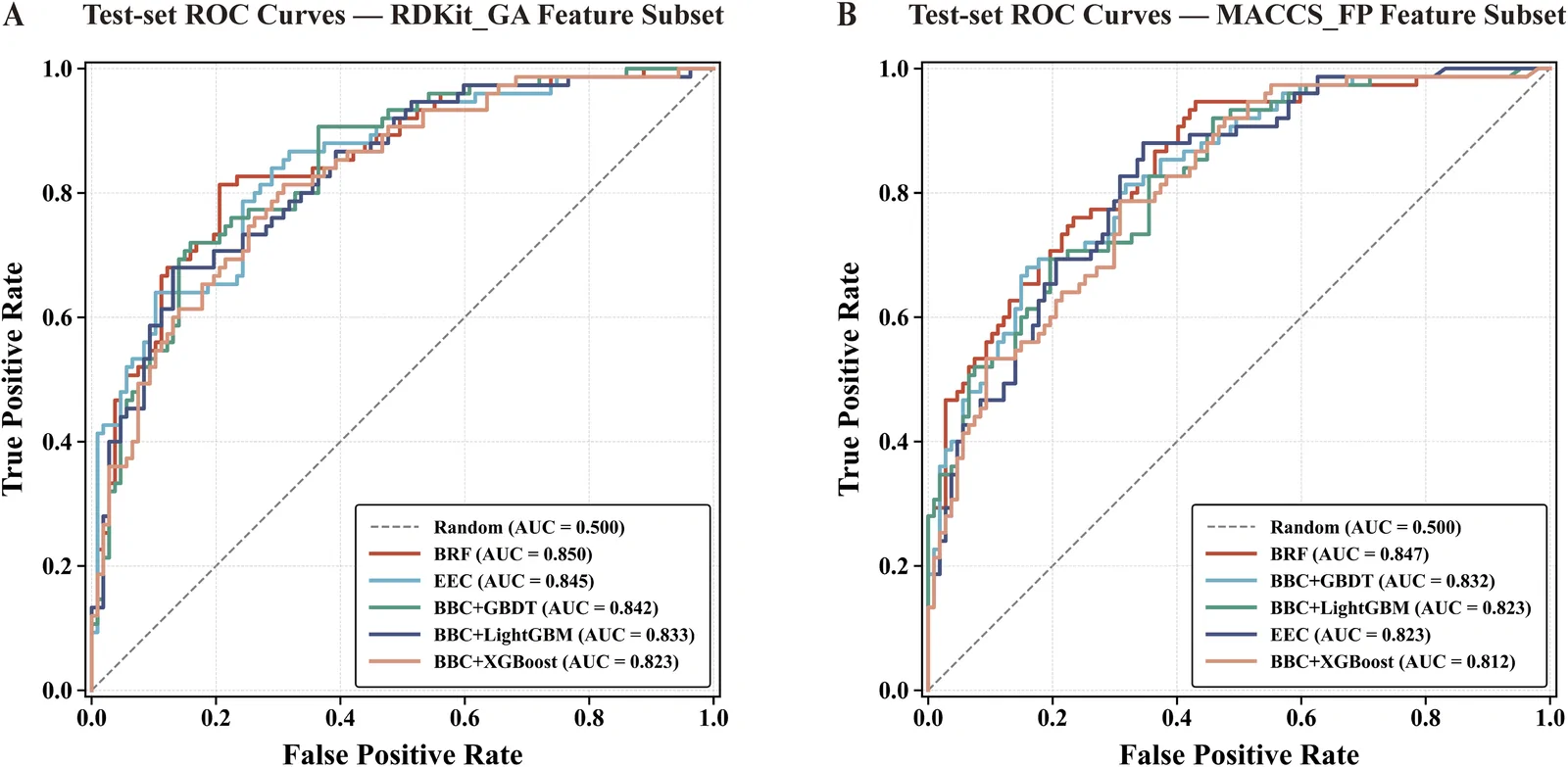
ROC curves on the test set for the five classification models trained on the two feature subsets. (A) RDKit_GA feature subset; (B) MACCS_FP feature subset.

For RDKit_GA, the standard and class-weighted baselines generally favored specificity over detection of the NCDIA-positive class: nine of the ten baseline models yielded mean SEN values ≤0.700, and only class-weighted XGBoost exceeded this level (0.711 ± 0.103), which remained lower than that of BRF (0.749 ± 0.101). Standard GBDT achieved the highest mean cross-validation MCC (0.541 ± 0.117), but its modest advantage over BRF (0.515 ± 0.091) was small relative to the fold-to-fold variability and was accompanied by a pronounced sensitivity – specificity imbalance (SEN = 0.663 ± 0.124 and SPE = 0.864 ± 0.038, compared with 0.749 ± 0.101 and 0.768 ± 0.078 for BRF). Among the imbalance-aware models, BBC + GBDT also marginally exceeded BRF in MCC (0.518 ± 0.090 *vs.* 0.515 ± 0.091), but showed lower sensitivity (0.711 ± 0.103) and a larger absolute SEN–SPE difference (0.092 *vs.* 0.019). RDKit_GA_BRF therefore provided a better balance among MCC, sensitivity, and class-wise performance under the prespecified selection criteria and was retained as the final descriptor-based model.

For MACCS_FP, all ten standard and class-weighted baselines showed greater specificity than sensitivity, with mean SEN ranging from 0.619 to 0.675 and mean SPE from 0.793 to 0.855, indicating a general tendency to favor the NCDIA-negative class. Standard RF achieved the highest baseline MCC, which was nearly identical to that of BRF (0.506 ± 0.109 *vs.* 0.503 ± 0.109). However, BRF provided substantially higher sensitivity (0.752 ± 0.075 *vs.* 0.659 ± 0.079), slightly higher BA (0.753 ± 0.055 *vs.* 0.746 ± 0.053), and a markedly smaller absolute SEN–SPE difference (0.003 *vs.* 0.175). Among the imbalance-aware models, EEC achieved slightly higher sensitivity than BRF (0.770 ± 0.079 *vs.* 0.752 ± 0.075), but had a lower MCC (0.487 ± 0.072) and a larger SEN–SPE difference (0.047). Considering MCC together with sensitivity and class-wise balance, BRF was retained as the final fingerprint-based model.

To further assess the stability of hyperparameter optimization conditional on the selected feature subsets, stratified nested cross-validation with five outer folds and five inner folds was conducted for the two selected BRF models using the 724-compound training set. Within each outer fold, BRF hyperparameters were optimized by five-fold inner cross-validation using MCC as the objective and 250 Hyperopt/TPE evaluations, after which the optimized model was refitted on the outer-training partition and evaluated on the corresponding outer-validation partition. The previously selected RDKit_GA and MACCS_FP feature subsets were retained in this analysis.

Across the outer folds, RDKit_GA_BRF achieved ROC-AUC = 0.829 ± 0.038, BA = 0.743 ± 0.040, SEN = 0.753 ± 0.088, SPE = 0.734 ± 0.068, and MCC = 0.483 ± 0.079. The corresponding values for MACCS_FP_BRF were 0.810 ± 0.034, 0.729 ± 0.045, 0.719 ± 0.103, 0.738 ± 0.046, and 0.455 ± 0.088, respectively (Table S7). Although modestly lower than the conventional 10-fold cross-validation estimates, both models retained moderate discrimination and broadly balanced sensitivity and specificity, indicating that predictive performance remained broadly consistent under fold-wise hyperparameter optimization.

Following training-set model selection, the two BRF classifiers were fitted on the complete training set and evaluated on the held-out test set. RDKit_GA_BRF achieved ROC-AUC = 0.850 (95% CI 0.791–0.903), BA = 0.799 (0.739–0.857), SEN = 0.813 (0.720–0.893), SPE = 0.785 (0.710–0.860), and MCC = 0.591 (0.472–0.706). MACCS_FP_BRF achieved ROC-AUC = 0.847 (0.788–0.899), BA = 0.761 (0.699–0.824), SEN = 0.747 (0.653–0.840), SPE = 0.776 (0.692–0.850), and MCC = 0.518 (0.396–0.643) (Table 4).

In paired bootstrap comparisons, RDKit_GA_BRF showed higher SEN [difference = 0.200 (95% CI 0.107–0.293)] and BA [0.062 (0.009–0.120)] than standard GBDT, while MACCS_FP_BRF showed higher SEN than standard RF [0.080 (0.013–0.147)]. The corresponding MCC-difference confidence intervals included zero [0.098 (−0.009 to 0.208) and 0.011 (−0.082 to 0.109), respectively]. These results indicate that BRF primarily improved the detection of NCDIA-positive drugs and class-wise balance, while no clear difference in MCC was observed relative to the strongest baseline models. Complete paired comparisons are provided in Table S8.

An ECFP4-Tanimoto similarity-weighted *k*-nearest-neighbour baseline was also evaluated using the same fixed training – test split, with *k* = 5 selected by training-set 10-fold cross-validation. On the held-out test set, this baseline achieved ROC-AUC = 0.787 (95% CI 0.718–0.851), BA = 0.717 (0.647–0.783), SEN = 0.667 (0.560–0.773), SPE = 0.766 (0.682–0.841), and MCC = 0.433 (0.294–0.568) (Table S9). Both selected BRF models showed higher point estimates across all evaluated metrics, particularly sensitivity and MCC, indicating better held-out performance than this simple local chemical-similarity baseline.

### 3.4. Applicability domain coverage of the held-out test set

To determine whether the held-out test compounds fell within each model's defined applicability domain, the ADs of the two final retained models—RDKit_GA_BRF and MACCS_FP_BRF—were characterized using the Euclidean Applicability Domain procedure described in Section 2.5, with normalized mean-distance scores calibrated separately against the training data in each feature space.

For both the RDKit_GA_BRF and MACCS_FP_BRF models, every drug in the held-out test set (*n* = 182) returned a normalized mean-distance score within the 0–1 interval, and consequently none was flagged as out-of-domain. This result indicates that all held-out compounds fell within the defined feature-space domains of the corresponding training data under both molecular representations, consistent with the broad overlap between the training and test sets in *M*_W_ − ALogP space shown in Fig. 2A. Accordingly, none of the test-set predictions was classified as extrapolative by the AD criterion, and the held-out performance metrics reported above characterize model performance for compounds within these defined applicability domains.

### 3.5. Structural interpretation of the SHAP-identified top features

Having confirmed that all held-out compounds fell within the defined applicability domains of both BRF models, we next examined the chemical features associated with these predictions. SHAP attributions calculated for the held-out test set were used to rank the inputs of each model, and the five most influential features were selected for detailed interpretation for the descriptor-based (RDKit_GA_BRF) and fingerprint-based (MACCS_FP_BRF) models (Fig. 4 and Table 5).

**Fig. 4:**
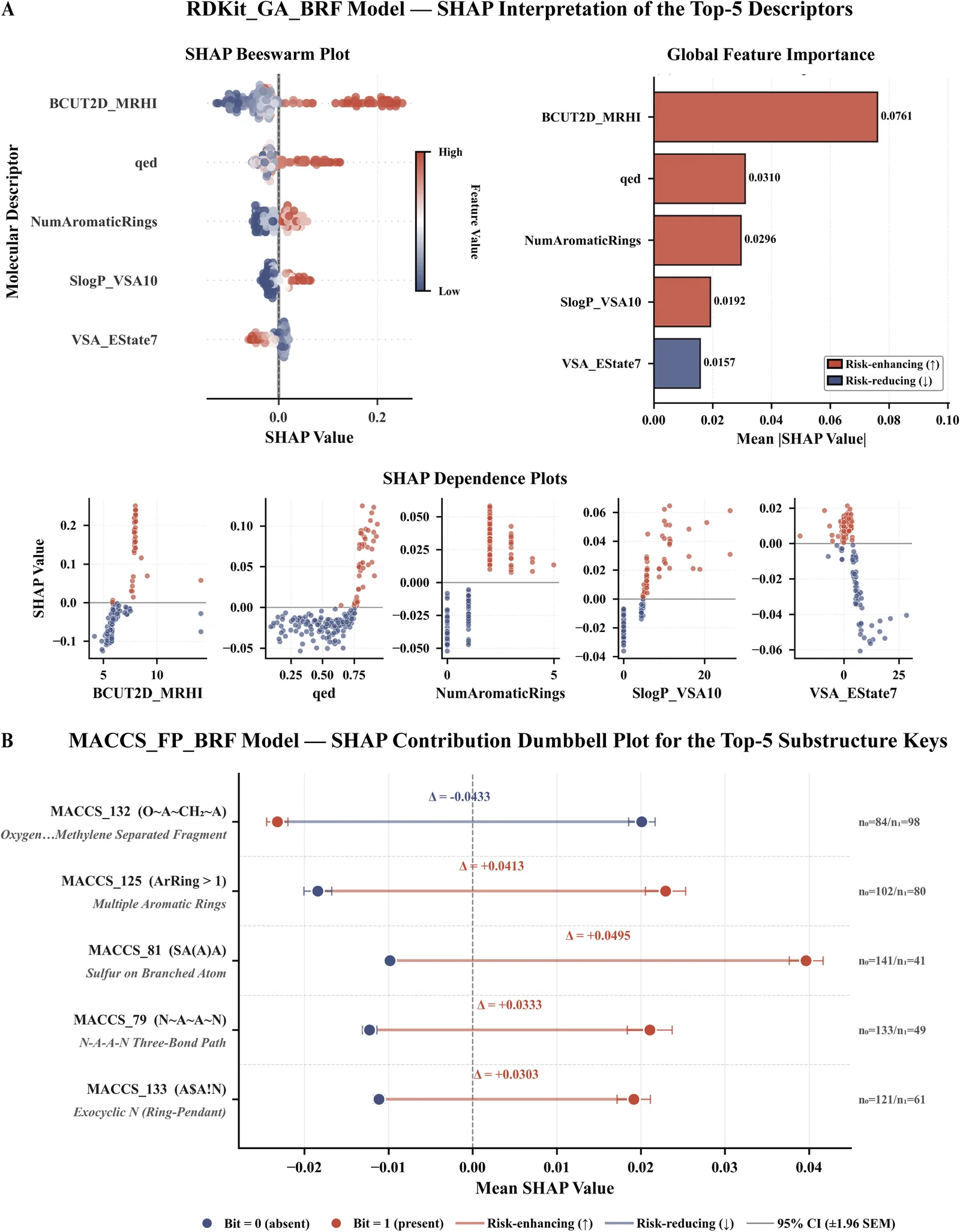
SHAP-based interpretation of the two BRF models for NCDIA risk prediction; all SHAP values were computed on the test set. Throughout, red and blue encode different quantities across the sub-panels—feature value, direction of effect, SHAP sign, or substructure presence—as defined in the panel descriptions below. (A) RDKit_GA_BRF model—SHAP interpretation of the Top-5 descriptors. SHAP beeswarm plot: each point is one test-set compound, its horizontal position the SHAP value, and its color the descriptor value from low (blue) to high (red). Global feature importance: descriptors ranked by mean absolute SHAP value, with bars colored by the net direction of effect—risk-enhancing (red, positive mean SHAP) or risk-reducing (blue, negative mean SHAP). SHAP dependence plots: for each of the five descriptors the SHAP value is plotted against the descriptor value, with points colored by the sign of the SHAP contribution (red, positive; blue, negative); a horizontal line marks SHAP = 0, and the plots reveal the threshold or monotonic shape of each feature's effect. (B) MACCS_FP_BRF model—SHAP contribution dumbbell plot for the Top-5 substructure keys. The five MACCS keys with the highest mean absolute SHAP value on the test set are shown, ordered top-to-bottom by decreasing importance and labeled with the MACCS index, its SMARTS abbreviation, and chemical meaning. Because each key is binary, the test compounds divide into two groups: for every key the blue dot marks the mean SHAP value across compounds in which the substructure is absent (Bit = 0) and the red dot the mean across those in which it is present (Bit = 1), where a positive SHAP value indicates a contribution that raises the predicted NCDIA probability. The connecting line, with its value annotated above, reports the contribution shift (*Δ* = mean SHAP_present − mean SHAP_absent)—the average change in the key's SHAP contribution on going from non-carrier to carrier—and is colored red when this shift is risk-enhancing (*Δ* ≥ 0) or blue when risk-reducing (*Δ* < 0); a larger |*Δ*| reflects a substructure whose presence more strongly reorients the model's prediction. The dashed vertical line marks SHAP = 0, so a key whose blue and red dots fall on opposite sides of it reverses the sign of its mean contribution between the two groups. Error bars are 95% confidence intervals (±1.96 standard error of the mean (SEM)), and *n*_0_/*n*_1_ on the right axis give the numbers of non-carrier and carrier compounds, which determine the precision of the two group estimates.

**Table 5:** Top-five SHAP-ranked features in the two selected BRF models for NCDIA prediction

Model	Importance rank	Feature	Source	Description	Mean absolute SHAP value
RDKit_GA_BRF	1	BCUT2D_MRHI	RDKit	Incorporates atomic molar refractivity in the Burden matrix - returns the highest eigenvalue	0.0761
	2	qed	RDKit	Quantitative estimate of drug-likeness	0.0310
	3	NumAromaticRings	RDKit	The number of aromatic rings in a molecule	0.0296
	4	SlogP_VSA10	RDKit	Sum of van der Waals surface areas of atoms with logP contribution in [0.4, 0.5)	0.0192
	5	VSA_EState7	RDKit	Sum of E-state indices of atoms whose van der Waals surface-area contribution falls in [6.07, 6.45)	0.0157
MACCS_FP_BRF	1	MACCS_132	MACCS	Oxygen⋯methylene separated fragment	0.0218
	2	MACCS_125	MACCS	Multiple aromatic rings	0.0204
	3	MACCS_81	MACCS	Sulfur on branched atom	0.0166
	4	MACCS_79	MACCS	N–A–A–N three-bond path	0.0147
	5	MACCS_133	MACCS	Exocyclic N (ring-pendant)	0.0139

A complementary robustness analysis was conducted using the training set to examine the reproducibility of the SHAP results. For both final models, all five leading SHAP features were ranked within the corresponding Top-20 lists in at least five of the six settings comprising FP and the five feature-selection strategies, and all five remained within the Top-20 across all ten cross-validation folds. The mean pairwise Spearman correlations across folds were 0.958 for the RDKit_GA_BRF model and 0.916 for the MACCS_FP_BRF model, while the corresponding mean Top-20 Jaccard indices were 0.701 and 0.812, respectively. These findings support the stability of the principal training-set SHAP rankings under the perturbations evaluated (Table S10). The five leading RDKit_GA_BRF features were identical and appeared in exactly the same rank order in the training and test sets. For MACCS_FP_BRF, the first four features were identical and appeared in the same order, whereas the fifth-ranked feature changed from MACCS_135 in the training-set analysis to MACCS_133 in the test-set analysis, indicating substantial overall consistency between the two datasets.

With the robustness of these leading features established, we examined their structural interpretation in the context of reported bioactivation mechanisms. Although the two feature representations have no variables in common, their leading features highlighted several overlapping chemical themes. The features separate into those flagging bioactivation-prone toxicophores (sulfur-containing pharmacophores, multiple aromatic rings, nitrogen-bearing/arylamine cores) and those encoding chemical-space context (aliphatic content, drug-likeness). Fig. 5 illustrates putative bioactivation routes relevant to these structural themes using representative test-set drugs, together with the contrasting aliphatic region associated with lower NCDIA-positive predictions; each is examined in turn below.

**Fig. 5:**
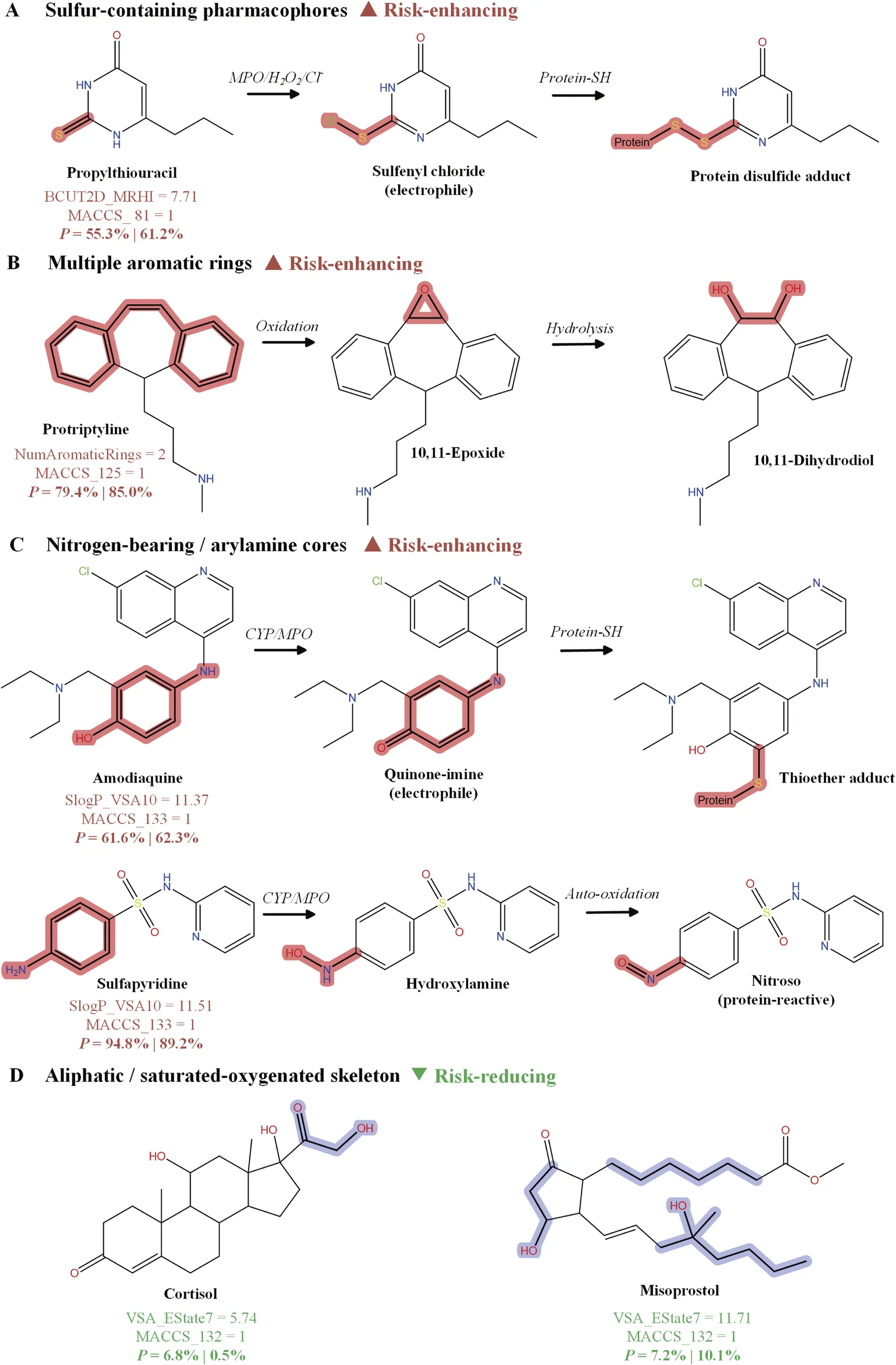
Proposed bioactivation mechanisms for the SHAP-identified NCDIA structure-toxicity themes, illustrated with representative test-set drugs. Reactive centers and risk-enhancing motifs are shaded coral; risk-reducing motifs blue. For the three risk-enhancing themes, true-positive drugs are shown with representative reported metabolic transformations or proposed bioactivation pathways: (A) sulfur-containing pharmacophores—propylthiouracil, whose oxidation by activated neutrophils or the MPO/H_2_O_2_/Cl^−^ system yields PTU-disulfide, propyluracil-2-sulfinate, and propyluracil-2-sulfonate; a PTU-derived sulfenyl chloride has been proposed as a possible intermediate; (B) multiple aromatic rings—protriptyline, with reported oxidation of the central 10,11-double bond to form a 10,11-epoxide; a 10,11-dihydrodiol metabolite has also been reported; (C) nitrogen-bearing/arylamine cores—CYP/MPO oxidation of amodiaquine's *para*-aminophenol to a quinone-imine and of sulfapyridine's free arylamine to hydroxylamine/nitroso metabolites. (D) The risk-reducing aliphatic/oxygenated region—correctly classified NCDIA-negative examples, cortisol and misoprostol—whose structures lack the specific risk-associated motifs highlighted by the present models. Each drug is annotated with its top SHAP feature value(s) and predicted NCDIA probabilities (RDKit_GA_BRF|MACCS_FP_BRF). The depicted transformations are literature-supported or proposed pathways and do not necessarily establish the mechanisms responsible for NCDIA for the specific drugs shown.

#### 3.5.1. Sulfur-containing pharmacophores: a cross-model risk signal

BCUT2D_MRHI is the largest eigenvalue of the Burden matrix constructed using Wildman–Crippen atomic contributions to molar refractivity (MR) on its diagonal, with its value influenced by both atomic contributions and molecular connectivity.^45^ It ranked first among RDKit features and was associated with higher NCDIA-positive predictions above a threshold value of approximately 7.04 (Fig. 4A). In the held-out test set, all 32 true-positive compounds above this threshold contained at least one sulfur atom. Given the relatively high atomic MR contributions of certain sulfur atom types compared with many common carbon, nitrogen, and oxygen atom types,^46^ this observation is consistent with sulfur contributing to elevated BCUT2D_MRHI values in these compounds. This sulfur-associated pattern was further supported by the dataset-level functional-group analysis (Fig. S3), which showed that thioether groups were significantly enriched among NCDIA-positive compounds (14.6% *vs.* 2.2%, BH-FDR-adjusted *p* < 0.001). This enrichment provides complementary dataset-level evidence for the association between sulfur-containing chemotypes and NCDIA-positive annotations. RDKit also includes multiple fragment descriptors representing specific sulfur-containing functional groups and heterocycles, such as sulfides, thiols, thiocarbonyls, sulfones, sulfonamides, thiazoles, and thiophenes. Unlike these fragment descriptors, which quantify individual structural motifs, BCUT2D_MRHI integrates atomic MR contributions and molecular connectivity across the entire molecular graph. In the final GA-selected RDKit feature subset, fr_SH, fr_sulfone, and fr_thiazole were retained, whereas several other sulfur-related fragment descriptors were not (Table S4). This may reflect differences in the structural information captured by these descriptors and their contributions within the multivariate model, rather than an absence of association between the excluded sulfur-containing motifs and NCDIA. A complementary sulfur-associated signal was also identified in the MACCS model: MACCS_81 (sulfur on a branched atom) emerged as one of the strongest keys associated with higher NCDIA-positive predictions in the SHAP dumbbell plot (Fig. 4B); it is carried exclusively by sulfur-containing drugs, approximately three-quarters of which are NCDIA-positive, compared with roughly one-third of non-carriers.

The sulfur-containing compounds in the dataset span several drug classes associated with idiosyncratic toxicity, including β-lactams, sulfonamides and sulfones, thiazides, thioamides, phenothiazines and thioxanthenes, as well as other thioether-, thiazole-, and thiophene-containing scaffolds. Sulfur-containing pharmacophores can undergo metabolic or neutrophil myeloperoxidase (MPO)-mediated oxidation, although the pathways are compound-dependent. For propylthiouracil (PTU), the thiourea moiety has been described as a structural alert whose oxidation may generate reactive metabolites implicated in blood dyscrasias.^47^ Oxidation by activated neutrophils and the MPO/H_2_O_2_/Cl^−^ system yielded PTU-disulfide, propyluracil-2-sulfinate, and propyluracil-2-sulfonate; a PTU-derived sulfenyl chloride was proposed as a possible intermediate (Fig. 5A).^47,48^ These PTU-derived species may contribute to PTU-associated blood dyscrasias, including agranulocytosis, potentially through direct cytotoxicity to neutrophils or myeloid progenitors and/or covalent protein binding, although the specific protein-reactive species remains unresolved.^47,48^ For drugs containing a thiophene scaffold, ticlopidine is another example: oxidation by activated neutrophils generated a reactive metabolite, and mass spectrometric evidence from the HOCl system suggested that this intermediate was a thiophene-*S*-chloride, which may contribute to ticlopidine-induced agranulocytosis.^49^ Critically, in arylamine-containing sulfonamides and β-lactams, the reactive center is not necessarily sulfur itself—toxicity may instead involve arylamine N-oxidation or covalent protein binding mediated by the β-lactam ring, respectively^4,8,50^—so sulfur is a shared structural hallmark of these classes rather than necessarily being the bioactivation site.

These associations are not deterministic: approximately one-quarter of MACCS_81 carriers were NCDIA-negative. Moreover, among the held-out test-set compounds with BCUT2D_MRHI values above approximately 7.04, some true-negative compounds contained iodine, bromine, or phosphorus rather than sulfur. This is consistent with the non-specific nature of BCUT2D_MRHI, which reflects both atom-specific MR contributions and molecular connectivity. Because some iodine and bromine atom types have higher atomic MR contributions than some sulfur atom types, elevated BCUT2D_MRHI values do not specifically indicate the presence of sulfur. Therefore, BCUT2D_MRHI is better interpreted as a non-specific descriptor associated with sulfur-enriched chemical space in this dataset. Nevertheless, the RDKit and MACCS models provide complementary evidence for an association between sulfur-containing structural features and NCDIA-positive annotations.

#### 3.5.2. Multiple aromatic rings and oxidative metabolism

NumAromaticRings—a simple count of aromatic rings and a recognized molecular property linked to compound developability^51^—shows a clean threshold effect in its SHAP dependence plot (Fig. 4A): counts of one or fewer contribute negatively, counts of two or more positively. The MACCS model recovers the identical fact—MACCS_125 (more than one aromatic ring) is exactly equivalent to a NumAromaticRings value of two or more in this dataset, and ranked among the top risk-enhancing keys of that model.

Aromatic rings can undergo cytochrome P450 (CYP)-mediated oxidation to form arene oxides; aromatic hydroxylation can also yield phenolic or catechol intermediates that may be further oxidized to quinones.^52^ In protriptyline, however, the reported epoxidation occurs at the central 10,11-double bond, and 10,11-epoxide and 10,11-dihydrodiol metabolites have been reported (Fig. 5B).^53,54^ Thus, protriptyline illustrates oxidative metabolism of a polycyclic scaffold rather than direct evidence of aromatic-ring epoxidation. The association between two or more aromatic rings and higher NCDIA-positive predictions therefore reflects a structural pattern in the dataset, rather than identifying a specific site of oxidative metabolism.

Two points merit note. The positive contribution does not scale with additional rings but saturates—and slightly attenuates—beyond two rings, so the model applies the feature as a near-binary switch rather than a graded effect. And because the presence of two or more aromatic rings is common among NCDIA-negative drugs, the feature is a non-specific indicator whose positive contribution is realized only alongside co-occurring descriptors. That two models based on non-overlapping molecular representations rank the same structural feature among their leading risk-enhancing features nonetheless supports the consistency of this signal across representation types.

#### 3.5.3. Nitrogen-bearing scaffolds and arylamine bioactivation

Two further risk-enhancing features—one RDKit descriptor (SlogP_VSA10) and one MACCS key (MACCS_133)—converge on nitrogen-bearing aromatic cores, while another MACCS key (MACCS_79) extends the signal to nitrogen-rich scaffolds more broadly.

SlogP_VSA10 is the van der Waals surface area (VSA) of atoms whose Wildman–Crippen logP contribution falls in the 0.4–0.5 band;^55^ despite its label it is essentially uncorrelated with overall lipophilicity. In this dataset those atoms are predominantly aromatic carbons bonded to nitrogen—approximately four-fifths of the relevant surface in above-threshold true positives—so the descriptor effectively quantifies the surface of aromatic C–N systems; SHAP showed a positive, graded association above a value of approximately 4.90 (Fig. 4A). The MACCS model points to the same chemistry through MACCS_133, an exocyclic ring-pendant nitrogen—a broad pattern encompassing arylamines, *N*-aryl amides, amino-heterocycles, and the acylamino groups of β-lactams. The dataset-level functional-group analysis provided independent support for this nitrogen-associated chemical-space pattern (Fig. S3). Aromatic nitrogen and aniline functionalities were significantly enriched among NCDIA-positive compounds (36.1% *vs.* 22.8% and 36.9% *vs.* 17.4%, respectively; BH-FDR-adjusted *p* < 0.001), indicating that nitrogen-containing aromatic environments highlighted by SHAP are broadly represented across the dataset rather than arising from a limited subset of compounds.

A chemically relevant subset shared by these two features is the free aniline-type primary arylamine (*e.g.*, sulfapyridine, dapsone, sulfacetamide): aromatic primary amines are a well-recognized bioactivation toxicophore class in agranulocytosis, oxidized by CYP and the neutrophil MPO system to hydroxylamine and nitroso reactive metabolites implicated in immune-mediated and/or directly cytotoxic granulocyte destruction (Fig. 5C).^4,8,52^ Other carriers more often bear nitrogen heteroaromatics or aminoheteroaromatics (the aminothiazole of cephalosporins, 4-aminoquinolines); these contribute aromatic C–N surface but are not themselves the reactive center—cephalosporin toxicity proceeds through β-lactam ring-opening and amodiaquine's through its *para*-aminophenol (Fig. 5C). Both features are thus structural correlates of the bioactivation propensity of aromatic C–N cores rather than specific toxicophores: the surface and substructure are not exclusive to positives (a substantial fraction of their carriers are NCDIA-negative), and their positive contribution is realized only through model integration. That an RDKit descriptor and a MACCS key built on non-overlapping representations both point to nitrogen-bearing aromatic cores is a further cross-model convergence.

A further key, MACCS_79 (the N–A–A–N three-bond path), is a more generic nitrogen-topology pattern—any two nitrogen atoms separated by three bonds—occurring across aliphatic diamine and aminoalkyl side chains, piperazines/diazines, and β-lactams. Unlike SlogP_VSA10 and MACCS_133, it does not selectively flag the free aniline-type arylamine toxicophore—its carriers are dominated by aminoalkyl/diamine chains, piperazines, diazines, and β-lactams rather than free arylamines—and is best read as a non-specific marker of nitrogen-rich, heterocyclic drug-like scaffolds; about two-thirds of its carriers are NCDIA-positive *versus* roughly one-third of non-carriers, its contribution again realized only alongside co-occurring descriptors.

#### 3.5.4. Drug-likeness (qed) as chemical-space context

Quantitative Estimate of Drug-likeness (qed) integrates molecular weight, lipophilicity, hydrogen-bond donors/acceptors, polar surface area, rotatable bonds, aromatic ring count, and structural alerts into a single drug-likeness score on the interval from zero to one;^56^ SHAP showed a positive association with NCDIA risk above a value of approximately 0.74 (Fig. 4A).

The true positives above this threshold share no common pharmacophore—they are a pharmacologically diverse set united only by being compact, moderately lipophilic, and limited in hydrogen-bonding capacity, comfortably within the oral-drug space defined by the Lipinski and Veber rules.^57,58^ What links them is their shared identity as conventional, systemically acting small-molecule drugs, which accords with the clinical profile of NCDIA—overwhelmingly caused by small-molecule drugs that reach circulating neutrophils and myeloid progenitors and are amenable to metabolic/MPO-mediated bioactivation under sustained dosing.^4^

The qed score is mechanistically non-specific: more than half of positive drugs fall below the threshold, and the association largely reflects dataset composition, the negative class being enriched in physicochemical outliers (contrast agents, atypically large/polar/lipophilic molecules, quaternary compounds) outside conventional drug space. The qed descriptor therefore encodes drug-type context rather than a toxicity mechanism—raising the prior that a molecule could cause NCDIA, while discrimination within drug-like space is left to other structural features such as BCUT2D_MRHI and the aromatic descriptors above.

#### 3.5.5. The aliphatic – saturated region as a risk-depleted chemical space

The remaining two features carry negative SHAP contributions and mark a region of chemical space that NCDIA-causing drugs sparsely populate, rather than flagging a toxicophore.

VSA_EState7, a descriptor from the EState-VSA family, is largest for molecules with extended aliphatic carbon skeletons; an atom-level decomposition shows its atoms to be predominantly aliphatic CH_2_/CH carbons, and SHAP showed a negative association above a value of approximately 3.67 (Fig. 4A). The MACCS model encodes the same axis through MACCS_132 (a methylene separated from an oxygen by one intervening atom), the only risk-reducing key among its top five.

Both describe the saturated/aliphatic-oxygenated region—fatty acids and prostaglandins, steroids, fat-soluble vitamins, sugars and polyols—chemotypes markedly under-represented among NCDIA-causing drugs (Fig. 5D).^2^ The negative association thus reflects a drug-type contrast rather than a protective mechanism: NCDIA risk concentrates among compact, aromatic, heteroatom-bearing conventional drugs that have low VSA_EState7. The direction is nevertheless consistent with the lower enrichment of the specific bioactivation-associated motifs highlighted above within these saturated/aliphatic chemotypes. That a continuous RDKit descriptor and a binary MACCS key independently reconstruct the same aliphatic-versus-aromatic divide is a further cross-model corroboration.

Taken together, the SHAP analyses show the two BRF models to be learning chemically sensible rules. Three risk-enhancing themes—sulfur-containing pharmacophores, multiple aromatic rings, and nitrogen-bearing/arylamine cores—each map onto documented routes of neutrophil injury (Fig. 5): the formation of reactive species, largely through CYP- and MPO-mediated bioactivation, that are cytotoxic to neutrophils and myeloid progenitors and/or haptenate their proteins to provoke immune-mediated destruction. Two further themes encode chemical-space context rather than toxicophores: qed locates a molecule within the conventional systemic small-molecule space in which NCDIA risk concentrates, whereas a large aliphatic – saturated skeleton (high VSA_EState7, or the MACCS_132 fragment) marks a comparatively risk-depleted region. Consistent with these SHAP-derived patterns, distributional analyses of the top-ranked descriptors and MACCS keys revealed significant differences between NCDIA-positive and NCDIA-negative compounds after false discovery rate correction (Fig. S4 and S5). These results provide complementary statistical support that the SHAP-highlighted features correspond to distinct chemical property distributions underlying the NCDIA-associated chemical space. The repeated convergence of the RDKit and MACCS models on similar chemistry, despite their non-overlapping feature representations, provides complementary cross-representation support for the identified structural associations and their chemical interpretation.

### 3.6. Case studies: linking SHAP attributions to bioactivation mechanisms in amodiaquine and sulfapyridine

To examine whether the model-derived attributions are chemically consistent with prior mechanistic evidence, we examined two correctly classified NCDIA-positive drugs with literature-reported bioactivation chemistry, interpreting each prediction through per-compound SHAP waterfall plots from both BRF models (Fig. 6). For each drug, we focus on how each model's five most important features contribute to its specific prediction, rather than on a compound-specific feature ranking.

**Fig. 6:**
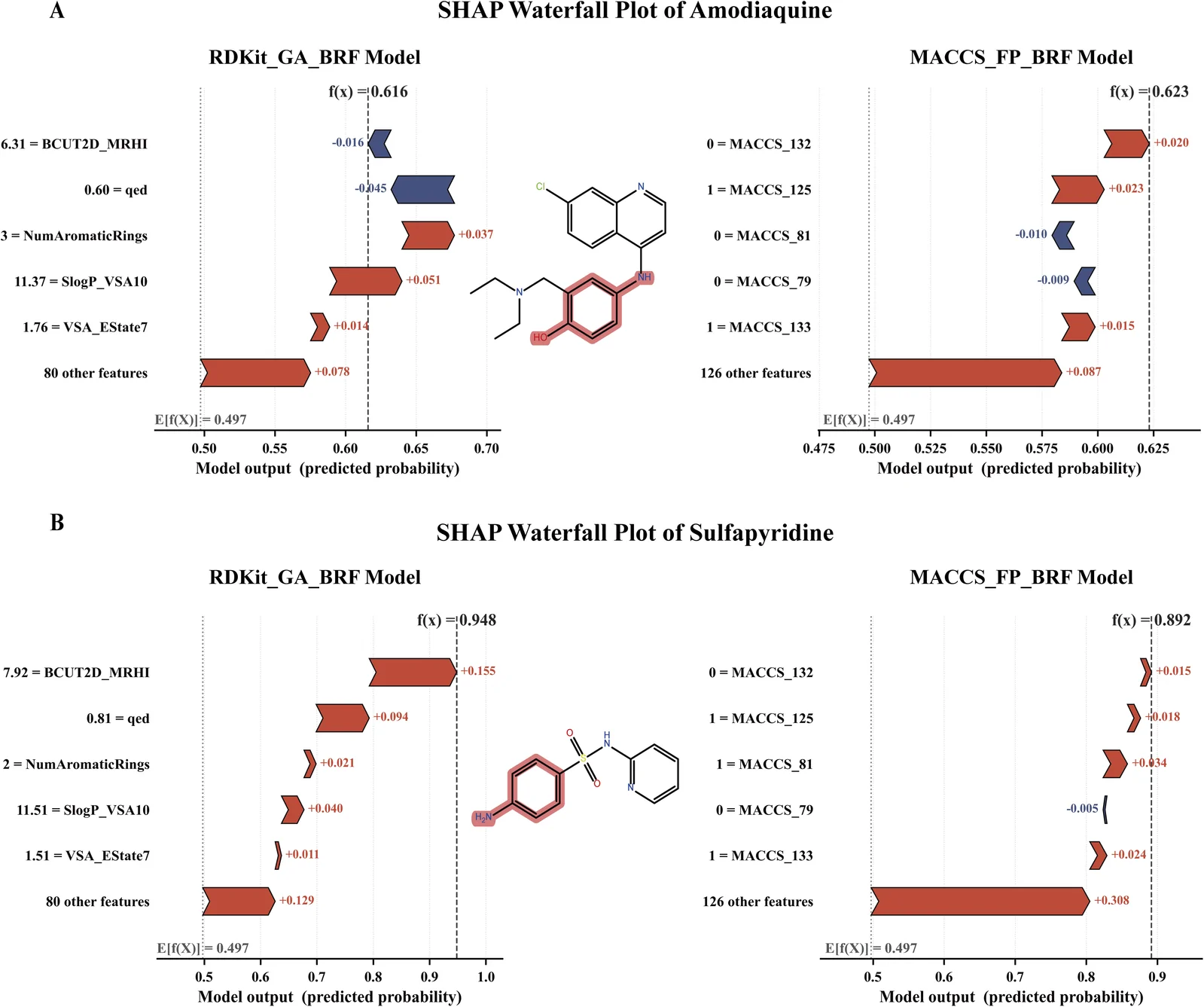
Per-compound SHAP waterfall plots for two case-study NCDIA-positive compounds correctly classified by both models: (A) amodiaquine and (B) sulfapyridine. For each compound, the prediction is decomposed for the RDKit_GA_BRF model (left) and the MACCS_FP_BRF model (right); the 2D structure is shown between the two plots, with red shading marking the bioactivation site (the *para*-aminophenol of amodiaquine in (A); the free primary arylamine of sulfapyridine in (B)). In each waterfall plot, the prediction starts from the model baseline *E*[*f*(*x*)] (bottom) and is built up additively by individual feature contributions to the final predicted probability *f*(*x*) (top), where *f*(*x*) > 0.5 corresponds to the NCDIA-positive class; red arrows denote positive SHAP contributions that raise the predicted NCDIA probability and blue arrows negative contributions that lower it. The value on each arrow is the feature's SHAP contribution, and the number to the left of the feature name is the compound's value for that feature (a descriptor value for the RDKit_GA_BRF model; a bit value, 0 = absent or 1 = present, for the MACCS_FP_BRF model). The five most globally important features of each model are shown individually and all remaining features are aggregated into a single “N other features” row.

Amodiaquine, a 4-aminoquinoline antimalarial, was assigned to the positive class by both models (Fig. 6A). The sulfur-pointing signal in each model contributed negatively—the molar-refractivity descriptor BCUT2D_MRHI (value 6.31, below its 7.04 risk threshold) in the RDKit model and the sulfur-on-branched-atom key MACCS_81 (absent) in the MACCS model—consistent with amodiaquine lacking the high-molar-refractivity atoms, sulfur in particular, that drive elevated BCUT2D_MRHI values. The positive prediction was instead driven by aromatic-system features: the aromatic ring count (NumAromaticRings) and the aromatic C–N surface descriptor (SlogP_VSA10) in the RDKit model, and the multiple-aromatic-ring (MACCS_125) and exocyclic ring-pendant nitrogen (MACCS_133) keys in the MACCS model. The absence of the risk-reducing oxygen-methylene key (MACCS_132) likewise pushed the MACCS prediction toward higher risk. Together these features delineate the polyaromatic, nitrogen-substituted scaffold whose *para*-aminophenol moiety is the established site of oxidative bioactivation: two-electron oxidation by hepatic cytochrome P450 and by the neutrophil MPO/H_2_O_2_ system generates the electrophilic amodiaquine quinone-imine (Fig. 5C), which covalently binds neutrophil proteins and produces the metabolism-dependent neutrophil cytotoxicity implicated in amodiaquine-induced agranulocytosis.^10,47,59^ Attribution and mechanism are therefore concordant, and the moderate positive model output is also consistent with the attribution pattern: lacking any sulfur reinforcement, the positive call rests on the aromatic theme.

Sulfapyridine, a sulfonamide and the moiety released from sulfasalazine by colonic bacteria,^60^ was assigned to the positive class with high confidence by both models (Fig. 6B), with the leading contributor in each being the sulfur-pointing feature—BCUT2D_MRHI in the RDKit model and MACCS_81 in the MACCS model—activated by the sulfonyl group. Mechanistically, however, the toxicity is more plausibly attributable to the free primary arylamine than to the sulfonyl sulfur. Sulfapyridine shows metabolism-dependent leukocyte toxicity requiring NADPH-dependent microsomal bioactivation,^61^ consistent with oxidation of its arylamine to a reactive hydroxylamine, which may further auto-oxidize to a protein-reactive nitroso species (Fig. 5C). An immune-mediated route is plausible: for arylamine benzenesulfonamides, such hydroxylamine/nitroso metabolites can act as haptens and elicit drug-specific T-cell responses,^62^ and the human leukocyte antigen (HLA)-locus association reported for sulfasalazine-induced agranulocytosis^60^ is consistent with this mechanism. A neutrophil-local route is also plausible but inferential: peroxidase/MPO-mediated oxidation of aromatic amines to reactive radical metabolites and protein free radicals on myeloperoxidase is established for related aniline-containing drugs,^63,64^ though not yet for sulfapyridine itself. The sulfonyl sulfur underlying the top SHAP attributions is therefore best interpreted as a sulfonamide-chemotype marker rather than the reactive center; the features that genuinely localize on the toxicophore—the aromatic C–N surface descriptor (SlogP_VSA10) and the ring-pendant nitrogen key (MACCS_133), encoding the aniline nitrogen and its aromatic ipso carbon—contributed in the same direction but with lower attribution.

Taken together, the two cases show that the model-derived SHAP attributions are chemically consistent with reported structure-bioactivation relationships. For amodiaquine, the aromatic and nitrogen-bearing features driving the positive prediction correspond to the polyaromatic scaffold containing the *para*-aminophenol site of oxidative bioactivation. For sulfapyridine, the correspondence is less direct—the largest attributions fall on the sulfonyl-sulfur features that mark the sulfonamide chemotype rather than the reactive center, while features related to the arylamine toxicophore contribute in the same direction but with lower weight. These case studies therefore illustrate structural associations that are consistent with prior mechanistic evidence, rather than demonstrating the underlying causal pathways.

### 3.7. ExNCDIA: an openly accessible web application for NCDIA prediction

To facilitate the practical application of ExNCDIA and to make it accessible to users without a computational background, the final models were deployed as a freely available web application (https://exncdia-predictor.streamlit.app/). The tool requires only a SMILES string as input and returns NCDIA class predictions from both the descriptor-based (RDKit_GA_BRF) and fingerprint-based (MACCS_FP_BRF) models. For every query compound, an applicability-domain assessment is also reported, indicating whether the molecule falls within the chemical space represented by the training data or constitutes an extrapolative prediction. The interface of the application is shown in Fig. 7.

**Fig. 7:**
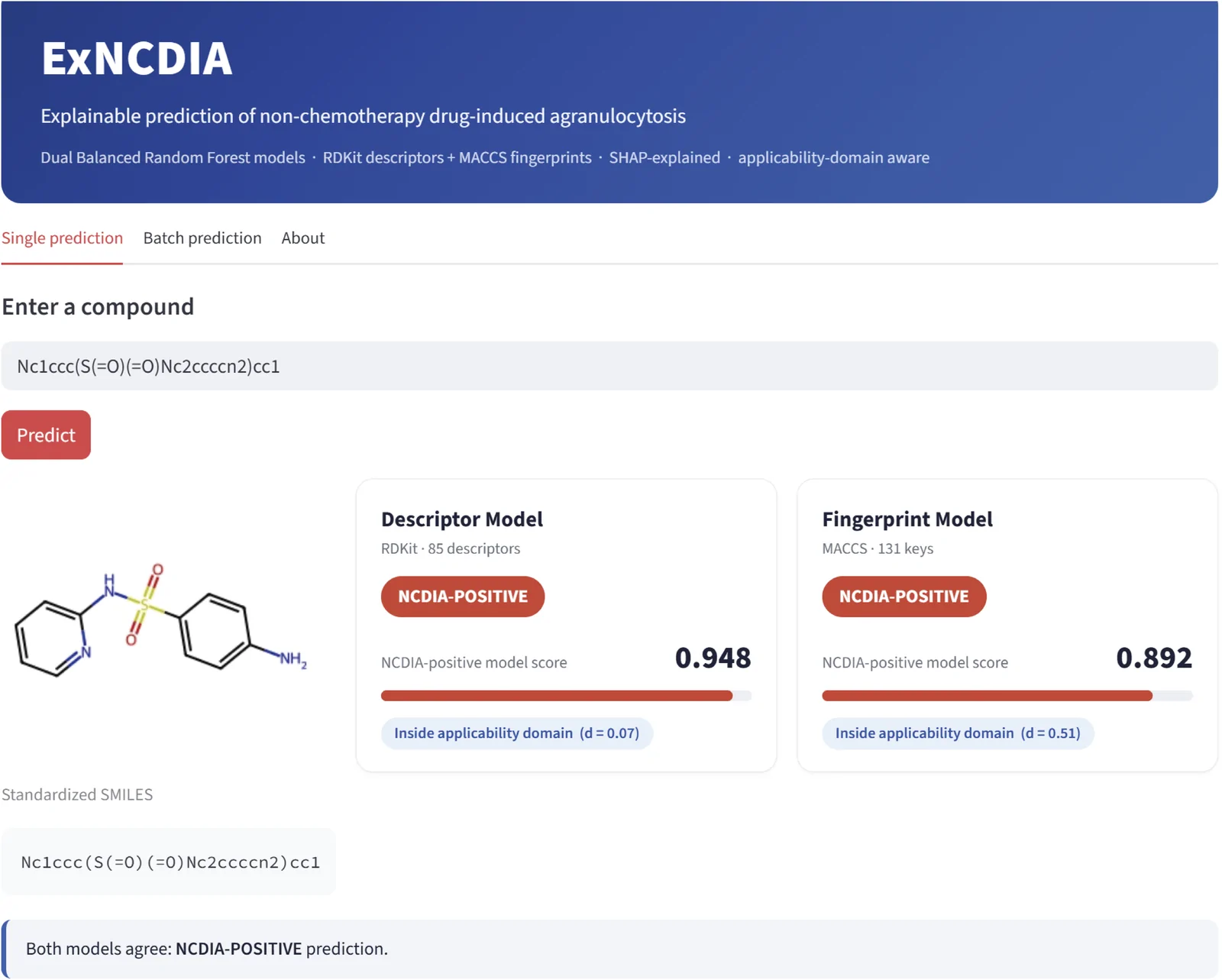
Interface of the ExNCDIA web application.

Beyond prediction, the application places particular emphasis on interpretability and usability. Each prediction is accompanied by a SHAP-based waterfall plot that quantifies and ranks the molecular features contributing to that specific model prediction, thereby providing interpretable, feature-level insight rather than a black-box score. The interface additionally supports batch prediction for the simultaneous evaluation of multiple compounds and allows the computed descriptors and fingerprints to be exported for further analysis. Implemented with the Streamlit framework, the application is accessible through a standard web browser and requires no local installation or programming expertise, enabling medicinal chemists to integrate NCDIA assessment into early-stage compound prioritization.

### 3.8. Strengths and limitations of this study

This study has several strengths. It tackles a clinically serious adverse reaction—non-chemotherapy drug-induced agranulocytosis—for which interpretable and reproducible models are still lacking, through a systematic pipeline that benchmarked four feature sets, five feature-selection methods (against a preprocessing-only baseline), and five imbalance-aware ensemble classifiers together with standard and class-weighted algorithm-matched baselines, with model selection guided by the imbalance-robust MCC and performance evaluated by 10-fold cross-validation and on a held-out test set. A distinctive feature of the study is the parallel use of two non-overlapping molecular representations. The descriptor-based RDKit_GA_BRF and fingerprint-based MACCS_FP_BRF models provided complementary views of molecular structure and converged on several chemically coherent structural patterns associated with NCDIA predictions across the two representation types. The combination of global and local SHAP analyses, feature-distribution analyses, and applicability-domain assessment further enabled the predictive results to be interpreted in the context of molecular properties and structural motifs. Finally, the freely accessible web predictor integrates model prediction, applicability-domain assessment, and compound-level SHAP explanation in a single interface, facilitating practical use in early-stage candidate prioritization and post-marketing risk assessment.

Several limitations should also be acknowledged. NCDIA is a largely idiosyncratic, multifactorial reaction shaped by dose, pharmacokinetics, and host factors such as immune and genetic predisposition—determinants that two-dimensional structure cannot capture; this intrinsic ceiling, together with a modest dataset (906 drugs) and binary labels compiled from heterogeneous sources, constrains the achievable accuracy and likely underlies the models' moderate predictive performance. Moreover, the NCDIA-negative designation reflects the absence of qualifying annotations in the sources examined, not the confirmed absence of risk; underreporting, limited market exposure, time on the market, and differences in regulatory labeling may therefore result in residual false-negative labels despite the broad SIDER exclusion dictionary and literature cross-check. Validation was internal only: because the test set was a stratified held-out set drawn from the same compound collection, its complete in-domain coverage is expected, and generalization to novel chemical space and real-world populations remains to be confirmed by external, prospective evaluation. Lastly, the SHAP-derived features are associative rather than causal—several are non-specific, recurring among NCDIA-negative drugs and contributing only in combination, and some (*e.g.*, BCUT2D_MRHI) serve as surrogates for the underlying chemistry; the proposed mechanisms are therefore literature-grounded hypotheses that warrant experimental confirmation.

## 4. Conclusions

In this study, we developed ExNCDIA, an explainable ensemble machine learning framework for predicting non-chemotherapy drug-induced agranulocytosis from fully open-source molecular representations. Built on an expanded, rigorously curated dataset of 906 drugs through an imbalance-aware pipeline, two balanced random forest models—one descriptor-based (RDKit_GA_BRF) and one fingerprint-based (MACCS_FP_BRF)—achieved well-balanced discrimination in training-set cross-validation and maintained balanced predictive performance on the held-out test set. Beyond prediction, SHAP-based interpretation revealed reproducible and chemically coherent structural associations across the two non-overlapping molecular representations. The complementary descriptor- and fingerprint-based analyses provided consistent information on molecular properties and substructural patterns associated with NCDIA predictions, while compound-level analyses of amodiaquine and sulfapyridine further illustrated how these model-derived features relate to established bioactivation chemistry. Together with applicability-domain assessment, these analyses provide a chemically interpretable framework for understanding model predictions at both global and individual-compound levels. Released as a freely accessible web predictor alongside the complete dataset, source code, and trained models, ExNCDIA provides a transparent and reproducible platform to support the early identification and evaluation of NCDIA-associated structural liability in drug discovery and post-marketing risk assessment.

## Author contributions

Xiaojie Huang: conceptualization, funding acquisition, investigation, methodology, project administration, supervision, writing – original draft. Shusong Jiang: data curation, methodology, visualization, writing – review & editing. Peineng Liu: data curation, methodology, resources, software, visualization, writing – review & editing.

## Conflicts of interest

There are no conflicts of interest to declare.

## Supplementary Material

RA-OLF-D6RA06159A-s001

RA-OLF-D6RA06159A-s002

## Data Availability

The data supporting this article have been included as part of the supplementary information (SI). The ExNCDIA web-based prediction platform is publicly accessible at https://exncdia-predictor.streamlit.app/. All datasets, trained models, and source code are openly available on GitHub at https://github.com/Huangxiaojie2024/ExNCDIA. Further inquiries can be directed to the corresponding author. Supplementary information is available. See DOI: https://doi.org/10.1039/d6ra06159a.
