# Supplementary material for: ExNCDIA: an explainable ensemble learning framework for predicting non-chemotherapy drug-induced agranulocytosis and interpreting structural associations
Source: RSC Adv. 2026 Nov 5. Online ahead of print. doi: 10.1039/d6ra06159a (PMC13736793; doi:10.1039/d6ra06159a)
Supplement: Supplementary file 1 [file RA-OLF-D6RA06159A-s002.pdf]

Supplementary Information (SI) for RSC Advances.
This journal is © The Royal Society of Chemistry

Supporting Figures

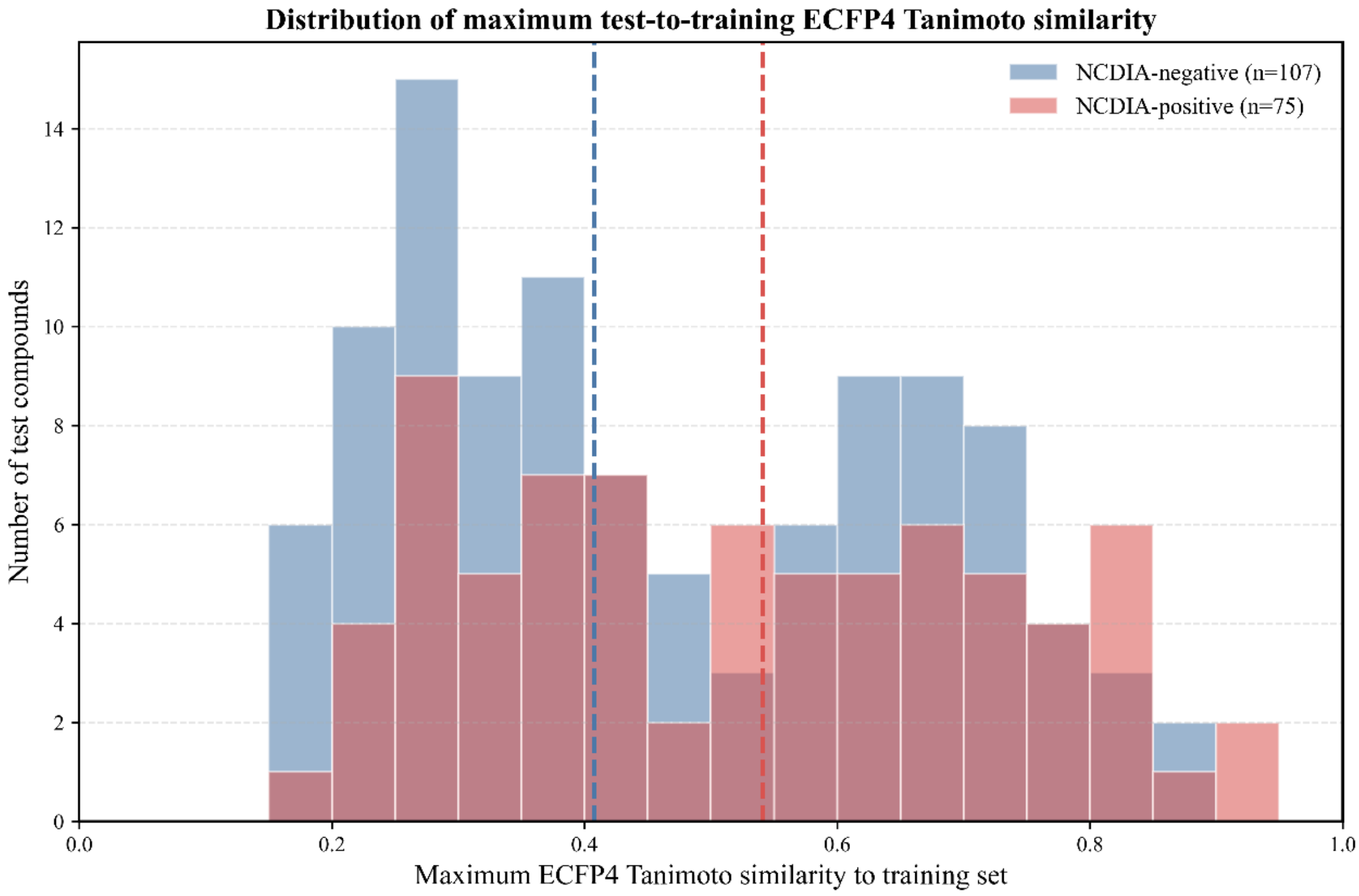

Fig. S1 Distribution of the maximum test-to-training ECFP4 Tanimoto similarity for the 182 held-out test compounds, stratified by NCDIA class. ECFP4 fingerprints were generated using a Morgan radius of 2 and 2048 bits without chirality. Dashed vertical lines indicate the median values for NCDIA-positive and NCDIA-negative test compounds.

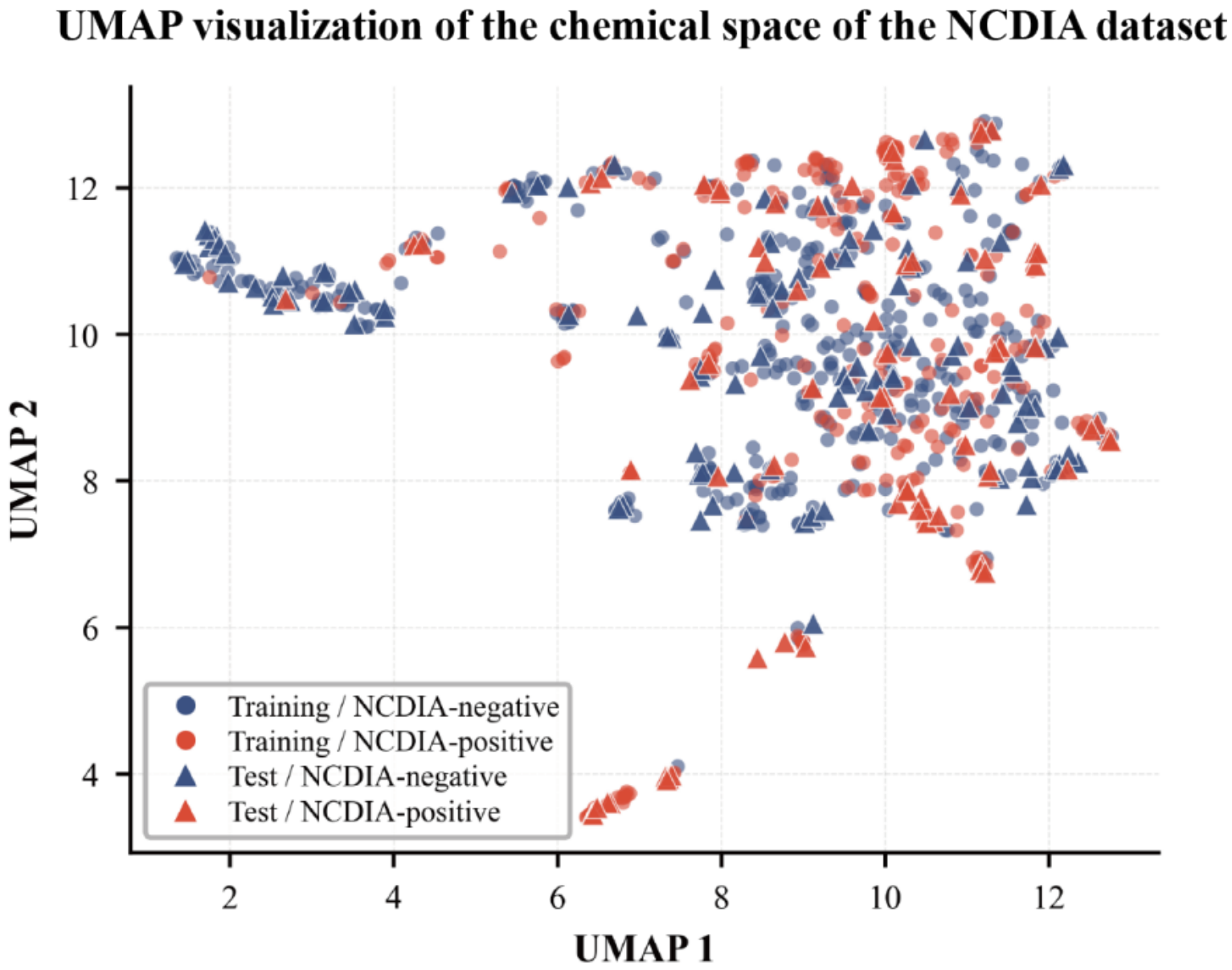

Fig. S2 Two-dimensional UMAP visualization of the 906 drugs in the NCDIA dataset based on pairwise Tanimoto distances (1 − Tanimoto similarity) derived from 2048-bit ECFP4 fingerprints. Each point represents one drug. Colors indicate NCDIA-negative (blue) and NCDIA-positive (red) compounds, while marker shapes distinguish training-set compounds (circles) from test-set compounds (triangles). UMAP was performed using 15 nearest neighbors and a minimum distance of 0.1.

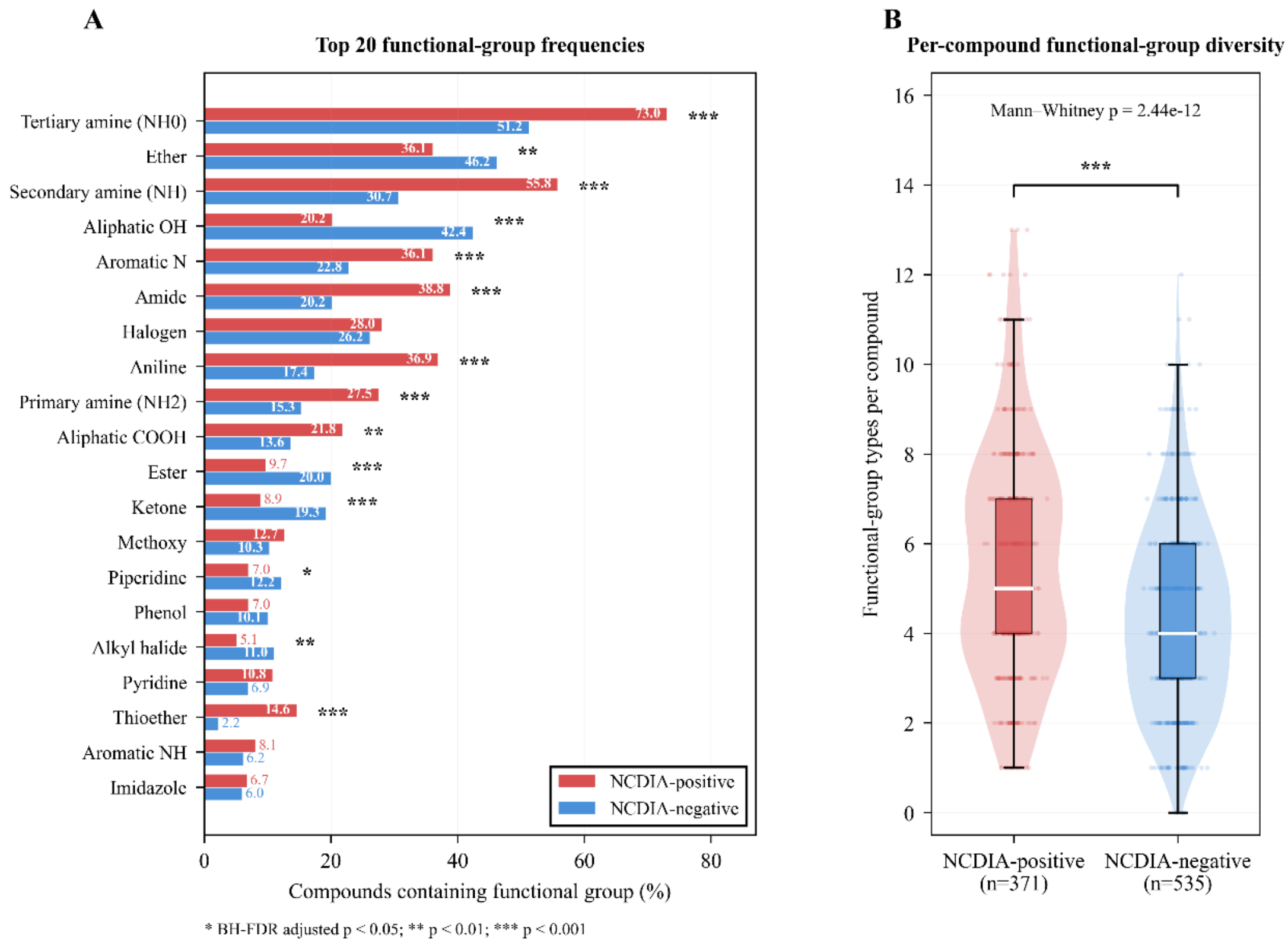

Fig. S3 Quantitative characterization of functional-group coverage in the NCDIA dataset. Functional groups were defined using a predefined panel of 51 RDKit fragment descriptors, with a group considered present when its corresponding fragment count was greater than zero. (A) Frequencies of the 20 most prevalent functional groups in NCDIA-positive (n = 371) and NCDIA-negative (n = 535) compounds, ordered by their overall prevalence in the complete dataset. Values within the bars indicate the percentage of compounds containing each group. Statistical significance was assessed using two-sided Fisher's exact tests with Benjamini–Hochberg false discovery rate (BH-FDR) correction across all 51 functional groups. Asterisks indicate BH-FDR-adjusted p values: *$p < 0.05$, **$p < 0.01$, and ***$p < 0.001$. (B) Distribution of the number of distinct functional-group types detected per compound. Violin plots show the overall distributions; boxes indicate the median and interquartile range, whiskers extend to 1.5 times the interquartile range, and points represent individual compounds. The two classes were compared using a two-sided Mann–Whitney U test ($p = 2.44 \times 10^{-12}$).

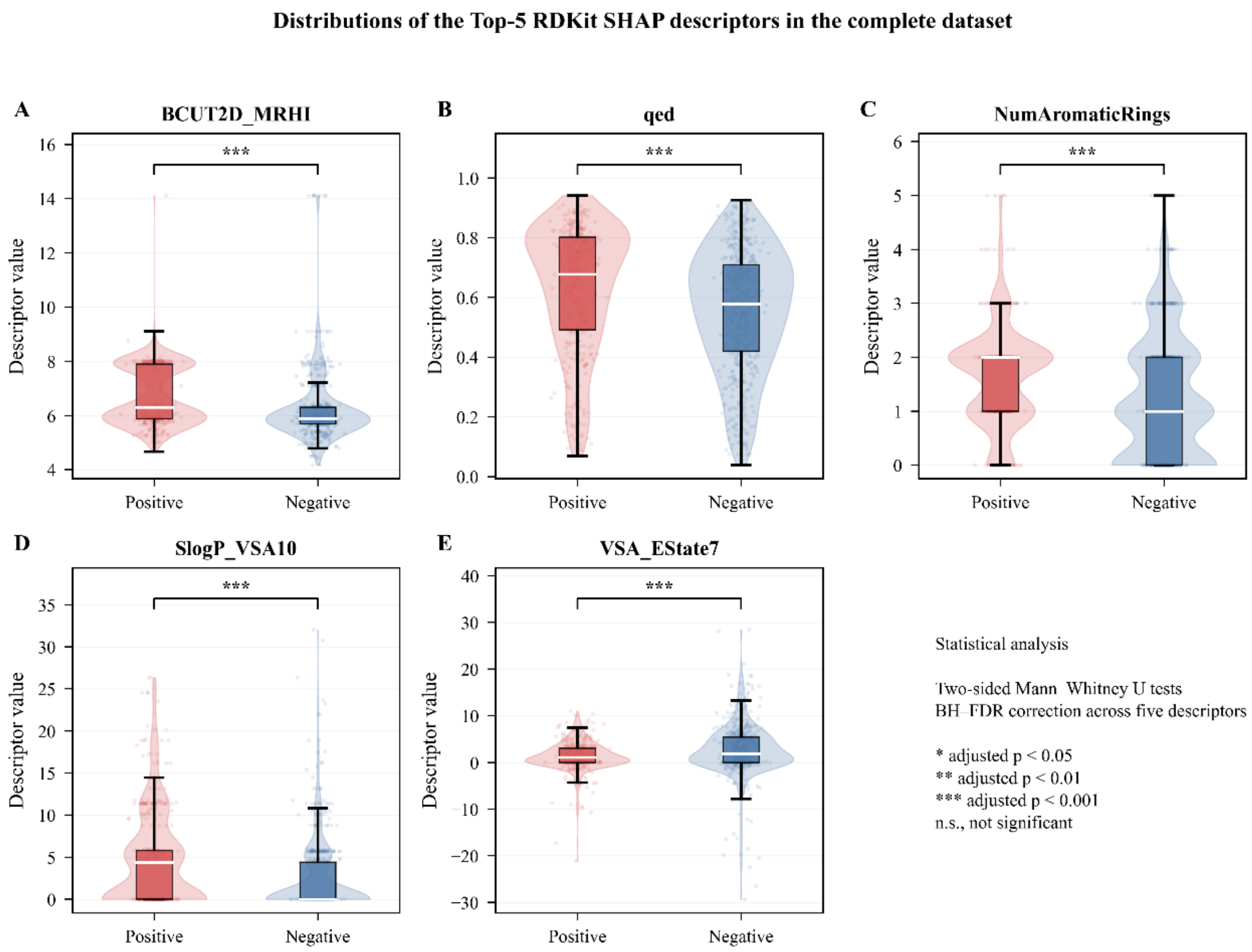

Fig. S4 Distribution of the top-ranked RDKit SHAP descriptors across the complete NCDIA dataset. The five descriptors with the highest mean absolute SHAP values identified during post hoc SHAP interpretation of the test set for the RDKit_GA_BRF model were subsequently examined across the complete dataset by comparing NCDIA-positive (n = 371) and NCDIA-negative (n = 535) compounds. Violin plots show the overall distributions, boxes indicate the median and interquartile range, whiskers extend to 1.5 times the interquartile range, and individual points represent compounds. Statistical comparisons were performed using two-sided Mann–Whitney U tests with Benjamini–Hochberg false discovery rate (BH-FDR) correction across the five descriptors. All five descriptors showed significant distributional differences between the two classes after correction. Asterisks denote BH-FDR-adjusted

significance levels: *p < 0.05, **p < 0.01, and ***p < 0.001.

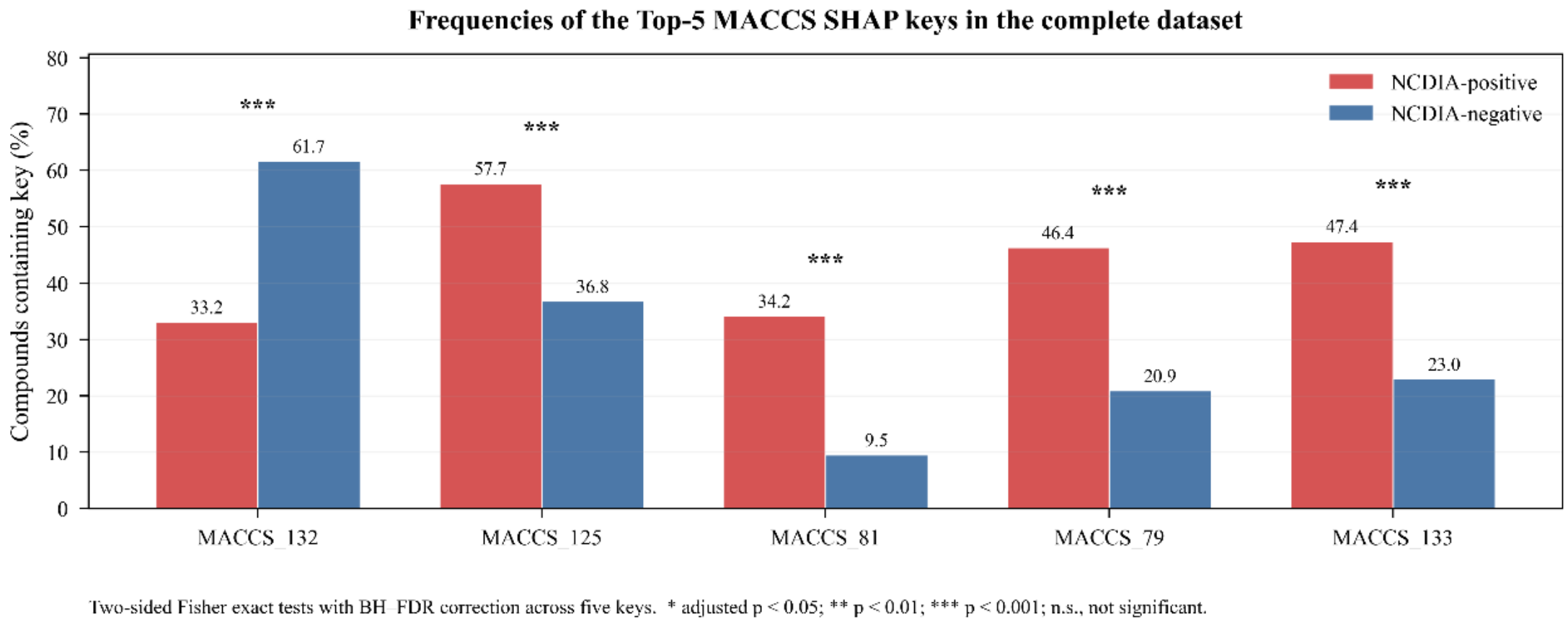

Fig. S5 Frequencies of the top-ranked MACCS SHAP keys across the complete NCDIA dataset. The five keys with the highest mean absolute SHAP values identified during post hoc SHAP interpretation of the test set for the MACCS_FP_BRF model were subsequently examined across the complete dataset by comparing NCDIA-positive (n = 371) and NCDIA-negative (n = 535) compounds. Values above the bars indicate the percentage of compounds containing each key within the corresponding class. Differences in key frequencies were assessed using two-sided Fisher's exact tests with Benjamini–Hochberg false discovery rate (BH-FDR) correction across the five keys. All five keys showed significant frequency differences between the two classes after correction. Asterisks denote BH-FDR-adjusted significance levels: *p < 0.05, **p < 0.01, and ***p < 0.001.
